# Integrated Transcriptomic and Proteomic Analyses Reveal Molecular and Cellular Responses to *INHA* Manipulation in Primary Yak Sertoli Cells

**DOI:** 10.3390/biology15211948

**Published:** 2026-10-29

**Authors:** Chao Chen, Mengli Cao, Liyan Hu, Xiaoyin Wang, Ziqiang Ding, Jiao Lin, Shaoke Guo, Jie Pei, Xian Guo

**Affiliations:** 1 Key Laboratory of Yak Breeding in Gansu Province, Lanzhou Institute of Husbandry and Pharmaceutical Sciences, Chinese Academy of Agricultural Sciences, Lanzhou 730050, China; chenchaocaas@163.com (C.C.); 821012450576@caas.cn (X.W.);; 2 Key Laboratory of Animal Genetics and Breeding on Tibetan Plateau, Ministry of Agriculture and Rural Affairs, Lanzhou 730050, China

**Keywords:** *INHA*, yak, Sertoli cell, transcriptomics, proteomics, cytokine signaling, apoptosis

## Abstract

*INHA* encodes the α-subunit of inhibin A and inhibin B and is involved in the regulation of male reproductive endocrinology; however, its cellular function in yak Sertoli cells remains poorly understood. In this study, primary yak Sertoli cells from 3-month-old donors were used to establish *INHA* overexpression and knockdown models, followed by RNA sequencing (RNA-Seq), directDIA quantitative proteomics, integrated transcriptome–proteome analysis, and functional assays. Donor-aware, false discovery rate (FDR)-controlled analyses identified 949 differentially expressed genes (DEGs) and 1388 differentially abundant proteins (DAPs) following overexpression, and 28 DEGs and 54 DAPs following knockdown. The two manipulations produced non-equivalent molecular responses under their respective experimental conditions. Functional enrichment analyses highlighted cytokine-related signaling, DNA replication and cell-cycle regulation, energy metabolism, and cell–matrix-related responses. TNF signaling was significantly enriched at both omics levels, whereas NF-κB signaling was significantly enriched at the transcriptomic level. Integrated analysis identified 98 molecules altered at both the mRNA and protein levels after *INHA* overexpression, of which 96 showed concordant changes. *CCL2*, *CCL5*, *IL1RL1*, *LIF*, *CTSC*, *HK2*, *FN1*, and *ITGA11* were prioritized as representative *INHA*-responsive candidates, while quantitative reverse transcription PCR (qRT-PCR) supported selected transcript-level changes. Flow cytometry showed that *INHA* overexpression was associated with increased apoptosis and a lower proportion of viable cells, whereas knockdown produced the opposite pattern. β-Tubulin immunofluorescence revealed qualitative differences in microtubule staining patterns. These results characterize molecular and cellular responses associated with *INHA* perturbation and prioritize candidate pathways and molecules for further investigation in yak Sertoli cells.

## Simple Summary

Yaks are important livestock in high-altitude regions, but the biological processes supporting male reproduction remain incompletely understood. We examined a gene that encodes one component of inhibin, a hormone involved in reproductive regulation. We used cultured Sertoli cells obtained from 3-month-old yaks; these cells support developing sperm cells. We increased or reduced the production of this gene’s protein product and examined changes in gene activity, protein levels, and cell survival. The molecular responses involved cell communication, processes related to cell division, energy use, and interactions between cells and their surroundings. Increased production was associated with more programmed cell death and fewer surviving cells, whereas reduced production showed the opposite pattern. Microscope images also suggested differences in the organization of fibers inside the cells. These findings identify molecules and biological processes for further investigation and provide a starting point for understanding the role of this gene in yak testicular supporting cells. Further research may contribute to knowledge of male reproductive health in yaks, which is relevant to sustainable livestock production and high-altitude farming communities.

## 1. Introduction

Yaks (*Bos grunniens*) are an important livestock species on the Qinghai-Tibet Plateau and in surrounding high-altitude regions, where they are adapted to hypoxia, low temperatures, and nutritional constraints [1,2]. Reproductive performance is crucial for maintaining and improving yak populations and is affected by testicular development, semen quality, and endocrine function in males [3]. Circulating testosterone and semen characteristics vary seasonally in yaks, reflecting the strong responsiveness of the male reproductive system to environmental and endocrine conditions [4]. However, the molecular regulation of testicular somatic cells in yaks remains less well-characterized than in many other livestock species [5].

Sertoli cells are key somatic components of the seminiferous epithelium and provide the structural and physiological environment required for germ cell development. Their functions include the formation of the blood–testis barrier, nutritional and metabolic support of developing germ cells, secretion of paracrine factors, regulation of the local immune environment, and remodeling of intercellular junctions [6,7]. Sertoli-cell function changes during testicular development and across stages of spermatogenesis, accompanied by transcriptional changes involving cell-cycle regulation, energy and retinoic acid metabolism, and barrier formation [6,8,9]. Currently, primary yak Sertoli cells can be obtained from testicular tissue and maintained in vitro, providing a useful experimental system for exploring gene function and cellular regulatory mechanisms [10,11].

In our previous studies, whole-transcriptome and full-length transcriptome sequencing were used to analyze gene-expression profiles in yak testicular tissue at different developmental stages. These analyses identified genes, non-coding RNAs, and regulatory pathways associated with testicular development and spermatogenesis [5,12]. Further investigation of development-associated expression patterns, together with available information on Sertoli cell function, identified *INHA* as a candidate gene of interest. However, the effects of altered *INHA* expression on yak Sertoli-cell physiology and associated molecular networks remain insufficiently characterized.

Inhibin A and inhibin B are heterodimeric glycoproteins belonging to the transforming growth factor beta (TGF-β) superfamily. The *INHA* gene encodes the α-subunit shared by inhibin A and inhibin B, which are formed through association of the α-subunit with the βA-subunit encoded by *INHBA* or the βB-subunit encoded by *INHBB*, respectively [13]. In most male mammals, inhibin B is primarily produced by Sertoli cells and contributes to negative-feedback regulation of pituitary follicle-stimulating hormone (FSH) secretion. Circulating inhibin B levels are therefore closely associated with Sertoli-cell function and spermatogenic activity [14,15]. Locally in the gonads, inhibin antagonizes activin signaling via betaglycan (TGFBR3) and activin type II receptors [16]; activin signaling, in turn, is involved in regulating Sertoli cell proliferation, differentiation, and blood–testis barrier function [17]. In chicken Sertoli cells, silencing *INHA* attenuated melatonin-induced proliferation, suggesting a potential local role for *INHA* in Sertoli-cell regulation, alongside the established endocrine functions of mature inhibins [18]. It remains unclear whether comparable responses occur in yak Sertoli cells and which molecular processes are involved.

Transcript abundance provides an incomplete representation of cellular responses because changes in mRNA abundance do not always translate into proportional changes in protein abundance [19,20]. Integrating transcriptomic and proteomic data can therefore offer a more comprehensive assessment of the molecular changes associated with *INHA* perturbation. In this study, we isolated and cultured primary yak Sertoli cells from 3-month-old donors, established *INHA* overexpression and knockdown models, and employed RNA sequencing, directDIA quantitative proteomics, and integrated transcriptomic–proteomic analysis to identify *INHA*-responsive changes and prioritize candidate molecules. Selected transcript-level changes were further assessed by quantitative reverse transcription PCR (qRT-PCR) for targeted confirmation. CCK-8 signal was summarized descriptively, apoptosis was assessed by Annexin V-FITC/PI flow cytometry, and microtubule staining patterns were examined qualitatively by β-tubulin immunofluorescence. The aim was to characterize the molecular and cellular responses associated with altered *INHA* expression and to provide experimental evidence for investigation of its role in immature yak Sertoli-cell biology.

## 2. Materials and Methods

### 2.1. Yak Sertoli Cell Isolation, Culture and Identification

Six healthy 3-month-old yaks raised under the same conditions in Hezuo City, Gannan Tibetan Autonomous Prefecture, were selected for the study. Testicular tissues were obtained following routine commercial slaughter for food production, unrelated to the present study. No animals were slaughtered specifically for this research. The left testis was obtained from each animal and processed independently during tissue preparation and cell isolation. Testicular tissues were thoroughly washed, cut into tissue pieces of approximately 2–4 mm^3^, and gradually cooled in a cryopreservation solution containing 80% Dulbecco’s modified Eagle medium (DMEM), 10% fetal bovine serum (FBS), and 10% dimethyl sulfoxide (DMSO), and then stored in liquid nitrogen.

For cell isolation, cryopreserved tissues were thawed, washed with prewarmed Dulbecco’s phosphate-buffered saline (DPBS), and finely minced. The tissue fragments were incubated with a digestion solution containing 2 mg/mL collagenase IV and 2 mg/mL hyaluronidase at 37 °C while shaking at 120 rpm for approximately 20 min. Following the first digestion, the tissue preparation was further digested with 0.25% trypsin under the same conditions for approximately 15 min to obtain a single-cell suspension. The resulting suspension was sequentially filtered through 70- and 40-μm cell strainers and centrifuged at 300× *g* for 5 min at 4 °C.

The cell pellet was resuspended in high-glucose DMEM containing 10% FBS and cultured at 37 °C in a 5% CO_2_ atmosphere. After an initial differential-adhesion period of 1.5 h, the non-adherent cell suspension was transferred to a new culture dish. Following cell attachment, the medium containing suspended cells was removed. The adherent cultures were maintained in serum-free high-glucose DMEM for 24 h and subsequently treated with 20 mM Tris–HCl hypotonic solution for approximately 5 min. The hypotonic solution was then removed, and the cells were returned to high-glucose DMEM containing 10% FBS. Brief differential trypsinization with 0.05% trypsin was used for further enrichment. This staged enrichment strategy followed principles used in previous yak Sertoli-cell studies [10,11,21].

Cells at passage 3 were assessed by morphological observation and immunofluorescence staining for GATA4 and vimentin. Cells were fixed with 4% paraformaldehyde for 20 min, permeabilized with 0.5% Triton X-100 for 20 min, and blocked with 3% bovine serum albumin for 30 min. Subsequently, cells were incubated separately with anti-GATA4 and anti-vimentin primary antibodies overnight, followed by incubation with the corresponding fluorophore-conjugated secondary antibodies for 1 h at room temperature in the dark. Cell nuclei were counterstained with 4′,6-diamidino-2-phenylindole (DAPI), and fluorescence images were acquired using a fluorescence microscope. Cell morphology, nuclear GATA4 staining, and cytoplasmic vimentin staining were evaluated together with the staged enrichment procedure to support the characterization of Sertoli-cell-enriched cultures.

Except for the pooled cell preparation used in the CCK-8 assay, Sertoli cells obtained from the six yaks were isolated, cultured, and experimentally treated independently, without pooling cells between animals. Each donor-derived Sertoli-cell culture was therefore defined as an independent biological replicate.

### 2.2. Construction of INHA Overexpression and Knockdown Models

The coding sequence of yak *INHA-201* (ENSBGRT00000033977.1, 1083 bp) was cloned into the pcDNA3.1(+) vector to construct the *INHA* overexpression plasmid (INHA-OE). The corresponding empty pcDNA3.1(+) vector served as the overexpression negative control (OE-NC). Both constructs were prepared by GenePharma (Shanghai, China). Four siRNAs targeting *INHA*, corresponding to positions 305, 572, 835, and 1062 of the target sequence, were designed and synthesized by GenePharma; the sequences are shown in Supplementary Table S1. A non-targeting siRNA was used as a negative control (si-NC). The four *INHA*-targeting siRNAs were initially screened under identical transfection conditions, and their effects on *INHA* mRNA levels were assessed by qRT-PCR. Among the four candidates, si-INHA-1062 showed the greatest reduction in *INHA* mRNA levels and was the sole siRNA used in downstream knockdown experiments.

Cells were transfected at about 80% confluence using INVI DNA RNA Transfection Reagent (Invigentech, Irvine, CA, USA). Transfection was performed in six-well plates containing 2.5 mL of antibiotic-free culture medium per well. For plasmid transfection, 10 μg of plasmid DNA was combined with 10 μL of transfection reagent per well. For siRNA transfection, 10 μL of a 20 μM siRNA stock solution (200 pmol) was combined with 10 μL of transfection reagent per well, giving a final siRNA concentration of approximately 80 nM. These doses were selected following preliminary dose optimization. The complexes were mixed by pipetting 10–15 times, incubated at room temperature for 10–15 min, and then added to the culture medium.

At 24 h after transfection, the medium was replaced with fresh complete culture medium. For endpoint analyses, cells were harvested at 48 h after transfection. Four experimental groups were included: OE-NC, INHA-OE, si-NC, and si-INHA. Transfection was assessed qualitatively by fluorescence microscopy in parallel cultures using a GFP-expressing reporter plasmid for plasmid transfection and a FAM-labeled control siRNA for siRNA transfection. The percentage of fluorescent cells and its variability among donors were not quantified. Changes in *INHA* expression were assessed separately by qRT-PCR and Western blotting.

### 2.3. RNA Extraction, cDNA Synthesis, and qRT-PCR

The treated cells were washed with pre-chilled PBS, and total RNA was extracted using TRIzol™ Reagent (Cat. No. 15596026; Invitrogen, Thermo Fisher Scientific, Waltham, MA, USA). RNA concentration and purity were determined using a NanoDrop™ 2000 spectrophotometer (Thermo Fisher Scientific), and samples with an A260/A280 ratio between 1.8 and 2.0 were used for subsequent experiments. For each sample, 1 μg of total RNA was reverse-transcribed using Hifair^®^ III 1st Strand cDNA Synthesis SuperMix for qPCR (gDNA digester plus, Cat. No. 11141; Yeasen Biotechnology, Shanghai, China). The reaction program comprised 42 °C for 2 min, 25 °C for 5 min, 55 °C for 15 min, and 85 °C for 5 min.

qRT-PCR was performed using the Hieff UNICON^®^ Universal Blue qPCR SYBR Green Master Mix (Cat. No. 11184; Yeasen Biotechnology) on a LightCycler^®^ 96 System (Roche Diagnostics, Basel, Switzerland). The amplification conditions consisted of an initial denaturation step at 95 °C for 2 min, followed by 40 cycles of 95 °C for 10 s and 60 °C for 30 s. A melting-curve analysis was conducted after amplification to assess amplification specificity. *ACTB* (β-actin) was used as the reference gene, and relative mRNA expression was calculated using the 2^−ΔΔCt^ method. The choice of *ACTB* was based on previous yak Sertoli-cell studies [11,21] and established laboratory practice. Preliminary experiments assessed primer amplification and melting-curve profiles; *ACTB* stability across the four treatment groups was not independently evaluated. For relative-expression calculations, OE-NC was used as the calibrator for the INHA-OE group, whereas si-NC served as the calibrator for the si-INHA group. Each group comprised six donor-derived biological replicates, with each replicate representing a separately maintained culture from an individual yak. The qRT-PCR measurements used separately cultured and treated cell preparations rather than the RNA aliquots used for RNA-Seq. qRT-PCR was used to assess *INHA* expression and to examine selected genes identified through RNA-Seq analysis. Primer sequences and relative-expression data for the six selected genes are listed in Supplementary Table S2.

### 2.4. Western Blotting

At 48 h after transfection, cells were washed with pre-chilled PBS and lysed on ice for 30 min in RIPA buffer containing 1% PMSF. Cell lysates were centrifuged at 12,000× *g* for 15 min at 4 °C, and the supernatants were collected. Total protein concentration was determined using the BCA assay. For each sample, 20 μg of total protein was separated by 12% SDS–PAGE and transferred onto a PVDF membrane. Membranes were blocked with 5% non-fat milk in TBST for 1 h at room temperature. The primary antibodies used were rabbit anti-INHA (1:12,000, Cat. No. 27331-1-AP; Proteintech, Wuhan, China) and rabbit anti-β-actin (1:5000, Cat. No. GB11001; Servicebio, Wuhan, China). Following overnight primary-antibody incubation at 4 °C and washing with TBST, membranes were incubated with HRP-conjugated goat anti-rabbit IgG (H+L) (1:15,000, Cat. No. 5220-0336; SeraCare, Gaithersburg, MD, USA) at room temperature for 1 h. Protein bands were visualized using enhanced chemiluminescence, and band intensities were quantified using ImageJ (version 1.54f). INHA protein abundance was normalized to β-actin. Three independent donor-derived biological replicates were analyzed per group. Uncropped raw Western blot images are shown in Figure S1.

### 2.5. CCK-8 Measurements, β-Tubulin Immunofluorescence, and Flow Cytometric Analysis of Apoptosis

Cells were seeded in 96-well plates, and CCK-8 measurements were performed at 12, 24, 36, and 48 h after transfection. At each time point, 10 μL of Enhanced Cell Counting Kit-8 reagent (CCK-8; Cat. No. BA00208; Bioss, Beijing, China) was added to each well and incubated at 37 °C for 2 h. Absorbance at 450 nm was then measured using a microplate reader. Untreated cell-control wells and cell-free blank wells were included in each experiment. Reference-normalized CCK-8 signal was calculated after correction for the background absorbance using the following formula:

Reference− normalized CCK−8 signal (%)=(Asample−Ablank)(Acontrol−Ablank)×100%

Definitions: *A*_sample_ represents the absorbance of the sample being evaluated; *A*_blank_ represents the absorbance of cell-free wells containing culture medium and CCK-8 reagent; *A*_control_ represents the absorbance of untreated, non-transfected cell-control wells at the corresponding time point.

At 48 h after transfection, cells grown on slides were washed with PBS, fixed with 4% paraformaldehyde for 20 min, and then permeabilized with 0.1% Triton X-100 for 10 min. After blocking the cells with 3% bovine serum albumin for 30 min, rabbit anti-β-tubulin primary antibody (Cat. No. 10094-1-AP; Proteintech, Wuhan, China) was added, and the samples were incubated overnight at 4 °C. After washing with PBS, cells were incubated with iFluor™ 594-conjugated goat anti-rabbit IgG (H+L) secondary antibody (1:1000; Cat. No. HA1122; HUABIO, Hangzhou, China) for 1 h at room temperature in the dark. After additional PBS washes, the samples were mounted using Antifade Mounting Medium with DAPI (Cat. No. MA0222; Meilunbio, Dalian, China), and images were acquired using a fluorescence microscope. The same image-acquisition parameters were maintained across all experimental groups. β-Tubulin immunofluorescence was used to observe qualitative differences in microtubule organization after *INHA* overexpression or knockdown.

At 48 h after transfection, cell apoptosis was evaluated using the Annexin V-FITC/PI Apoptosis Detection Kit (Cat. No. AP101; MultiSciences Biotech, Hangzhou, China). The 1× Binding Buffer was prepared by diluting the 5× stock solution with purified water. Approximately 1 × 10^6^ cells were collected from each sample, washed with 2 mL of PBS, and centrifuged at 300× *g* for 5 min. The resulting cell pellet was resuspended in 100 μL of 1× Binding Buffer and stained with 5 μL of Annexin V-FITC and 10 μL of propidium iodide (PI). Samples were incubated for 5–10 min at room temperature in the dark. Subsequently, 400 μL of 1× Binding Buffer was added, and the cell suspension was filtered through a 300-mesh cell strainer and analyzed using a BriCyte E6 flow cytometer (Mindray, Shenzhen, China).

Acquisition targeted more than 10,000 cellular events per tube. The cell population was gated using forward-scatter area (FSC-A) and side-scatter area (SSC-A) to select the main cell population and exclude events outside this gate, followed by singlet selection using FSC-A and forward-scatter height (FSC-H). Annexin V-FITC/PI quadrant analysis was performed on the gated singlets, and population percentages were expressed relative to this parent population. Annexin V^−^/PI^−^ (Q4), Annexin V^+^/PI^−^ (Q3), and Annexin V^+^/PI^+^ (Q2) cells were defined as viable, early apoptotic, and late apoptotic cells, respectively. Q1 represented Annexin V^−^/PI^+^ cells. Total apoptosis was defined as the sum of the proportions of early and late apoptotic cells (Q2 + Q3). Flow cytometry data were analyzed using MRFLOW v01.13.00.9306 and FlowJo v10.6.2 software. Three independent donor-derived biological replicates were analyzed per group, with each replicate representing a separately maintained cell culture from an individual yak.

### 2.6. RNA Sequencing and Bioinformatic Analysis

Six independent donor-derived biological replicates per experimental group were used in the RNA-Seq analysis. Cells from each donor were assigned to all four experimental groups, allowing donor-matched comparisons. Total RNA concentration and purity were assessed using a NanoDrop 2000 spectrophotometer (Thermo Fisher Scientific, Waltham, MA, USA), and RNA integrity was determined using an Agilent 2100 Bioanalyzer (Agilent Technologies, Santa Clara, CA, USA). Poly(A)+ mRNA was enriched using mRNA Capture Beads, and sequencing libraries were constructed with the Hieff NGS^®^ Ultima Dual-mode mRNA Library Prep Kit (Cat. No. 12309ES; Yeasen Biotechnology, Shanghai, China). After library quality assessment, 150-bp paired-end sequencing was performed on the Illumina platform (Illumina, San Diego, CA, USA) by Guangzhou Aozhi Biotechnology Co., Ltd. (Guangzhou, China). Across the 24 samples, sequencing generated 37.22–56.54 million raw reads per sample, with 36.82–55.89 million reads retained after quality filtering. Clean-read Q30 values ranged from 91.20% to 93.41%. Overall and unique genome-mapping rates were 89.52–91.28% and 77.27–79.21%, respectively, relative to the reads submitted for alignment. Sample-level quality metrics, expression matrices, the derived analysis-input matrix, and donor–treatment metadata are provided in Supplementary Table S3.

Raw sequencing reads underwent quality control using fastp v0.18.0 [22], and rRNA reads were removed using Bowtie2 v2.4.4 [23]. The resulting clean reads were aligned to the *Bos grunniens* reference genome (LU_Bosgru_v3.0, Ensembl release 110; GCA_005887515.1) using HISAT2 v2.1.0 [24]. Transcripts were reconstructed using StringTie v1.3.4, and expression was quantified using Salmon v1.10.3. Gene-level estimated-count and transcripts per million (TPM) matrices were used as inputs for downstream analysis.

A derived scaledTPM count-scale matrix was generated by multiplying each gene’s TPM by the sample-specific sum of exported estimated counts and dividing by the sample-specific sum of TPM values [25]. The resulting values were rounded to the nearest integer for input into PyDESeq2 v0.5.4 [26]. Overexpression and knockdown comparisons were analyzed separately, each comprising six donor-matched pairs. Genes with a total count below 10 across the 12 samples in a comparison were excluded. A negative-binomial model incorporating donor and treatment effects (~donor + condition) was fitted, and treatment effects were evaluated using Wald tests with Cook’s-distance filtering and independent filtering [27]. Multiple-testing correction was conducted using the Benjamini–Hochberg method, and genes with FDR < 0.05 and |log2 fold change| > 1 were defined as differentially expressed genes (DEGs).

Gene Ontology (GO) and Kyoto Encyclopedia of Genes and Genomes (KEGG) over-representation analyses were performed using the hypergeometric test. GO and KEGG assignments were obtained from the annotations accompanying the expression matrices. GO annotations were expanded to ancestor terms through is_a and part_of relationships using GO ontology release 15 June 2026. For each comparison and annotation category, the background comprised genes with valid differential-expression results and corresponding annotations. Terms or pathways with Benjamini–Hochberg-adjusted *q* < 0.05 were considered significantly enriched. Preranked gene set enrichment analysis (GSEA) was performed using GSEApy v1.3.1, with genes ranked by the Wald statistic from the donor-adjusted differential-expression model [28]. The analysis used 1000 gene-set permutations, a gene-set size range of 15–500, a weighting parameter of 1, and a random seed of 123. Gene sets with an absolute normalized enrichment score (NES) > 1, nominal *p* < 0.05, and FDR *q* < 0.25 were considered to meet the preset GSEA threshold, with FDR *q* < 0.05 indicating more stringent statistical evidence. Gene sets with nominal *p* < 0.05 but FDR *q* ≥ 0.25 were interpreted only as exploratory trends.

### 2.7. Proteomic Analysis

Six independent donor-derived biological replicates from each group were included in the proteomic analysis. Each donor contributed samples to all four experimental groups, allowing donor-matched comparisons. After quantifying total protein concentration using the BCA assay, denaturation, reduction, alkylation, trypsin digestion, and peptide desalting were performed using the iST Sample Preparation Kit (Cat. No. P.O.00027; PreOmics, Planegg, Germany). For each sample, 200 ng of peptides were analyzed by LC-MS/MS using an UltiMate 3000 LC system coupled with a timsTOF Pro 2 mass spectrometer (Bruker Daltonics, Bremen, Germany). Peptides were separated on a C18 column using a 60-min gradient, and mass spectra were acquired in diaPASEF mode over an m/z range of 349–1229.

Raw mass spectrometry data were analyzed using the library-free directDIA workflow implemented in Spectronaut 18 (Biognosys, Schlieren, Switzerland) against the *Bos grunniens* protein database from Ensembl release 110. Carbamidomethylation of cysteine was specified as a fixed modification, whereas oxidation of methionine was included as a variable modification. False discovery rates at both the precursor and protein levels were controlled at 1%. Protein quantification used identifications passing these FDR thresholds. After local normalization, protein abundances were determined using MaxLFQ.

Differential-abundance analysis used normalized protein abundances without missing-value imputation. Non-positive abundance values were treated as missing, and proteins with at least four complete donor-matched pairs in a comparison were retained. Proteins with fewer than four complete pairs were excluded from statistical testing in that comparison. Overexpression and knockdown comparisons were analyzed separately using two-sided paired Student’s *t*-tests on log2-transformed abundances. Benjamini–Hochberg correction was applied across all valid protein tests within each comparison. Fold changes (FCs) were calculated as 2 raised to the mean within-donor log2 abundance ratio. Proteins with (FC > 1.2 or FC < 1/1.2) and a Benjamini–Hochberg-adjusted q < 0.05 were defined as differentially abundant proteins (DAPs). An existing imputed abundance matrix was used only for sensitivity analysis, applying the same paired tests and effect-size and FDR thresholds, with six complete donor pairs required. Complete primary differential-abundance results, the normalized nonimputed abundance matrix, the imputed sensitivity-analysis matrix, and donor–treatment metadata are provided in Supplementary Table S4.

GO and KEGG over-representation analyses were performed using the hypergeometric test, with an FDR-adjusted *q*-value < 0.05 considered statistically significant. Annotation processing and multiple-testing correction followed Section 2.6, using comparison-specific backgrounds of proteins with valid differential-abundance results and corresponding annotations. Proteomic GSEA ranked proteins by the paired *t* statistic and used the same software, permutation number, gene-set size range, weighting parameter, random seed, and interpretation thresholds described in Section 2.6.

### 2.8. Integrated Transcriptomic and Proteomic Analysis

Transcriptomic and proteomic datasets were matched using yak gene identifiers, and the log2 fold change (log2FC) values and statistical results for the INHA-OE vs. OE-NC and si-INHA vs. si-NC comparisons were integrated separately. DEGs and DAPs were defined using the screening criteria specified for each omics dataset. Molecules meeting the differential criteria at both the transcript and protein levels were further classified as concordant or discordant based on the direction of change in both datasets. Pearson correlation coefficients were calculated across matched mRNA–protein pairs with finite log2FC values and valid differential-expression tests in both datasets. These correlations were used descriptively to summarize the overall relationships between transcript- and protein-level changes.

To compare the magnitude of effects across different omics datasets, log2FC values were scaled by the respective thresholds: 1 for transcripts and log2(1.2) for proteins. Molecules meeting the differential criteria in both datasets and showing concordant changes were ranked in descending order using min(|log2FC_RNA|, |log2FC_protein|/log2(1.2)), with gene identifiers used to resolve ties. For the heatmap, candidates from a selected list were included if they met the concordant differential criteria, and additional molecules were selected by rank to obtain 30 unique gene symbols. Signed threshold-scaled log2FC values were displayed. Paired mRNA–protein plots showed unscaled log2FC values for selected candidates and were not restricted to the significant overlap; protein effects failing the minimum complete-pair requirement were marked as unavailable. Candidate molecules were selected based on concordance between transcriptomic and proteomic changes, effect magnitude, and functional annotations relevant to Sertoli-cell biology. This selection was used solely for result visualization and biological interpretation and did not modify the statistical criteria or significance testing applied to either dataset. The complete ranked candidate list, unscaled and threshold-scaled effect sizes, figure-inclusion indicators for ranked candidates, and matched mRNA–protein results are provided in Supplementary Table S5.

Selected GO terms and KEGG pathways from the transcriptomic and proteomic analyses were paired using their corresponding term or pathway identifiers. Statistical significance was determined independently within each omics dataset using an FDR-adjusted *q*-value < 0.05. Hence, the presence of a matched GO term or KEGG pathway in both datasets was not considered evidence of significant enrichment at both molecular levels unless the significance threshold was independently met in each analysis.

### 2.9. Statistical Analysis

For assays other than CCK-8, biological replicates were defined as primary Sertoli-cell cultures derived from different yak donors and maintained and processed independently throughout the experiments. Each group included six biological replicates for RNA-Seq, proteomic analysis, and qRT-PCR. For Western blotting and flow cytometric analysis, three independent donor-derived cell cultures were randomly selected per group before the Western blot and apoptosis measurements, with sample size limited by assay costs. Selection was not based on the outcomes of these assays. For the CCK-8 assay, six technical replicate wells from a single pooled cell preparation were used per group at each time point, with separate plates used for different time points. Randomization concerned the selection of cultures for Western blotting and flow cytometry; no formal protocol for random treatment allocation or blinded assessment was documented.

Quantitative data are presented as mean ± standard deviation (SD). Statistical analysis of experimental data was conducted using GraphPad Prism 9.5.1 (GraphPad Software, La Jolla, CA, USA). For qRT-PCR, Western blotting, and flow-cytometric apoptosis assays, each treatment group was compared with its corresponding negative control using two-tailed unpaired Student’s *t*-tests, and *p* < 0.05 was considered statistically significant. Western blot tests used untransformed INHA/β-actin ratios, and flow-cytometry tests used population percentages. For Western blotting and flow cytometry, effect sizes were expressed as treatment-minus-control mean differences, with 95% confidence intervals calculated under the same equal-variance model. Flow-cytometry differences are reported in percentage points. These *p*-values and confidence intervals were not adjusted for multiple comparisons. CCK-8 measurements were summarized as mean ± SD across technical wells, with SD reflecting within-preparation well-to-well variation. No inferential statistical tests of treatment, time, or treatment × time interaction were performed for CCK-8 data.

Statistical procedures specific to the transcriptomic, proteomic, and enrichment analyses, including donor adjustment, paired testing, multiple-testing correction, and significance thresholds, are described in Section 2.6, Section 2.7 and Section 2.8. Pearson correlation coefficients were used descriptively to characterize associations between transcript- and protein-level log2 fold changes among matched molecules with valid statistical results and finite effect estimates. Individual Western blot ratios and flow-cytometry population percentages, together with exact *p*-values, mean differences, and 95% confidence intervals for these assays, are provided in Supplementary Table S5.

## 3. Results

### 3.1. Establishment of INHA Overexpression and Knockdown Models in Yak Sertoli Cells

**Figure 1:**
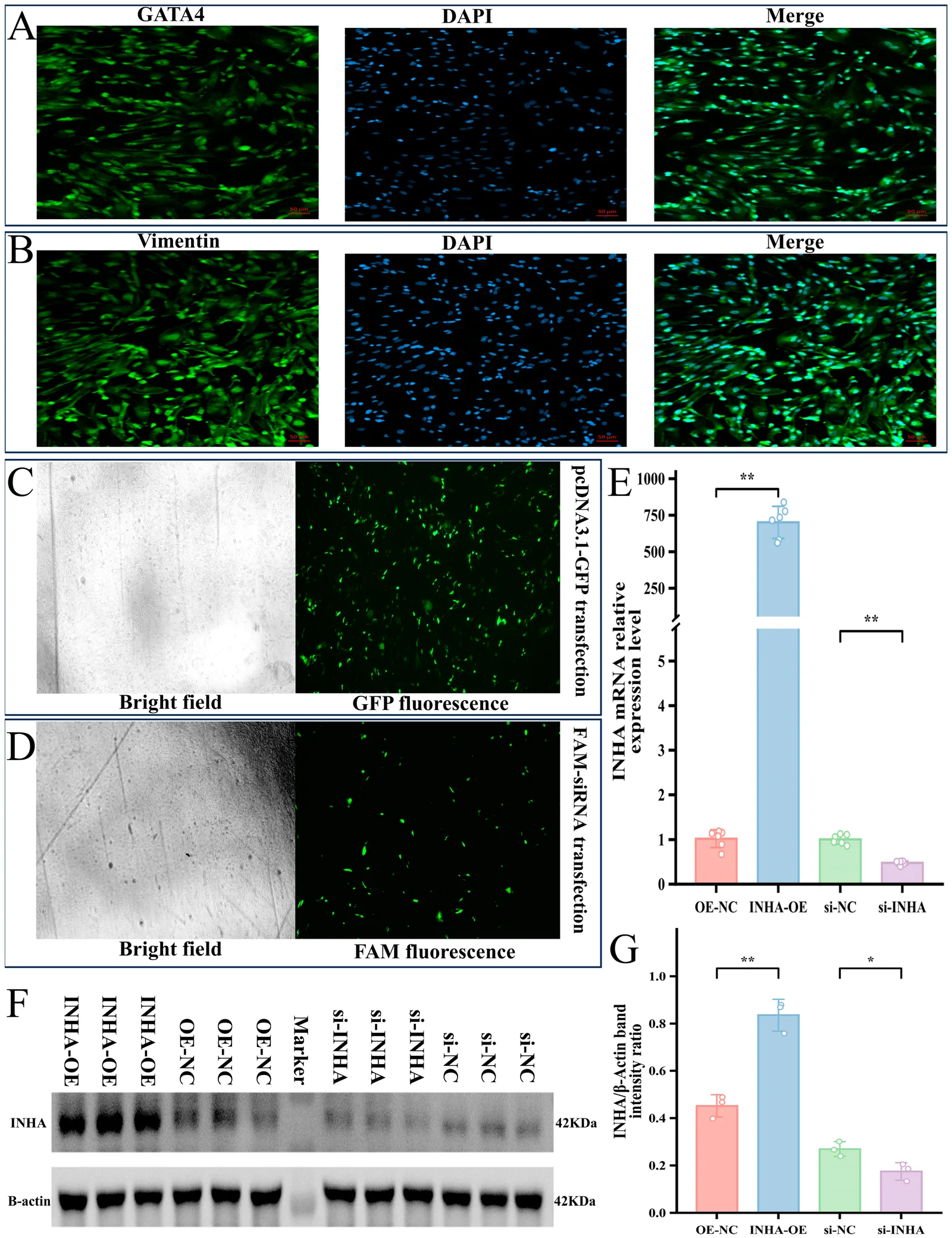
Characterization of primary yak Sertoli cells and validation of *INHA* overexpression and knockdown models. (**A**,**B**) Immunofluorescence staining of GATA4 and vimentin in isolated yak Sertoli cells. Nuclei were counterstained with DAPI. Scale bars: 50 μm. (**C**,**D**) Representative bright-field and fluorescence images of cells transfected with the pcDNA3.1-GFP reporter plasmid or FAM-labeled control siRNA. (**E**) Relative *INHA* mRNA expression determined by qRT-PCR (n = 6). (**F**) Representative Western blots of INHA α-subunit and β-actin; uncropped images are provided in Figure S1. (**G**) Densitometric quantification of INHA α-subunit abundance normalized to β-actin (n = 3). n denotes independent donor-derived biological replicates per group. Data are presented as mean ± SD. For E and G, each treatment was compared with its corresponding negative control using two-tailed unpaired Student’s *t*-tests. * *p* < 0.05 and ** *p* < 0.01.

Morphological evaluation and immunofluorescence staining were performed to characterize the isolated primary cells. As shown in Figure 1A,B, the cultured cells exhibited typical spindle-shaped or irregular morphology, with multiple processes characteristic of Sertoli cells. Immunofluorescence staining indicated positive signals for both GATA4 and vimentin. Specifically, GATA4 immunoreactivity was primarily localized to the nuclear region and overlapped with DAPI staining, whereas vimentin was mainly distributed throughout the cytoplasm in a distinct filamentous pattern. Together with the staged enrichment procedure described in Section 2.1, these morphological and immunofluorescence findings supported the characterization of the cultures as Sertoli-cell-enriched preparations. Exploratory image-level quantification of GATA4-associated nuclear fluorescence and vimentin-associated perinuclear fluorescence is presented in Figure S2.

To establish models of altered *INHA* expression, Sertoli cells were transfected with the *INHA* overexpression plasmid (INHA-OE) or *INHA*-targeting siRNA (si-INHA). GFP fluorescence was readily detected in cells transfected with the reporter plasmid, whereas FAM fluorescence was observed after transfection with the labeled control siRNA (Figure 1C,D), providing qualitative evidence of nucleic-acid delivery in parallel cultures.

The effects of *INHA* overexpression and knockdown were then assessed at both the mRNA and protein levels. qRT-PCR analysis revealed higher *INHA* mRNA expression in the INHA-OE group than in the OE-NC group (mean relative expression: 701.17 ± 109.65 versus 1.02 ± 0.20; *n* = 6 per group), corresponding to a 688.18-fold ratio of group means (*p* < 0.01), and lower expression in the si-INHA group than in the si-NC group (*p* < 0.01; Figure 1E). Western blotting showed corresponding changes in INHA α-subunit abundance, with higher INHA abundance in the INHA-OE group than in the OE-NC group (INHA/β-actin band-intensity ratio: 0.836 ± 0.067 versus 0.452 ± 0.047; *n* = 3 per group), corresponding to a 1.85-fold ratio of group means (*p* = 0.00126), and lower INHA abundance in the si-INHA group than in the si-NC group (*p* = 0.0281; Figure 1F,G). The treatment-minus-control differences in INHA/β-actin ratios were 0.384 (95% CI: 0.252–0.516) for overexpression and −0.094 (95% CI: −0.172 to −0.017) for knockdown. Individual ratios and complete statistical results are provided in Supplementary Table S5. Uncropped Western blot images are provided in Figure S1. These results supported the establishment of *INHA* overexpression and knockdown models in primary yak Sertoli cells for subsequent molecular and cellular analyses.

### 3.2. Transcriptomic Profiling Reveals Extensive Molecular Responses to INHA Manipulation

**Figure 2:**
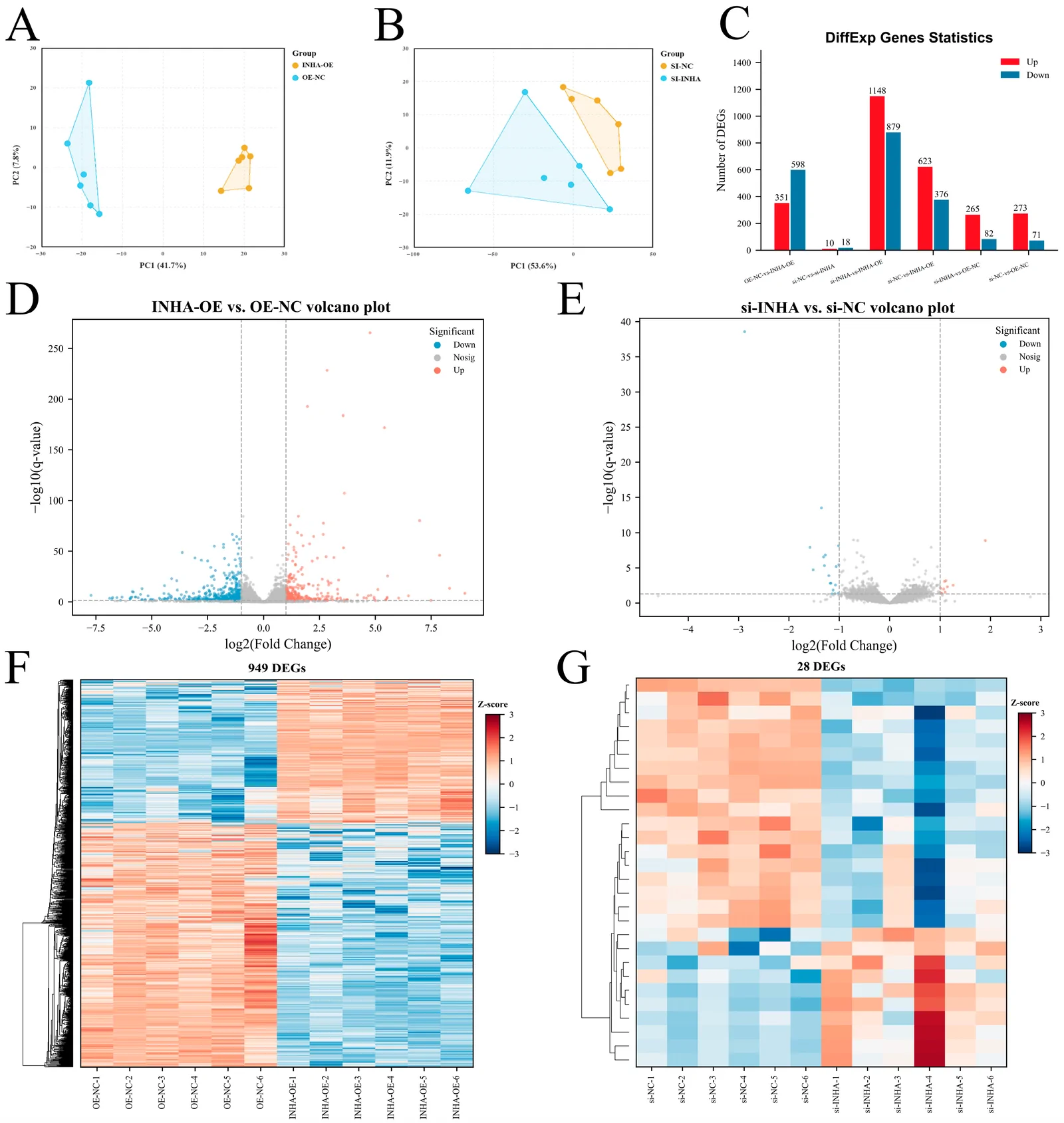
Transcriptomic profiling reveals global gene expression changes following *INHA* manipulation in yak Sertoli cells. (**A**,**B**) Principal component analysis (PCA) of RNA-Seq samples from *INHA*-overexpressing (INHA-OE), control (OE-NC), *INHA*-knockdown (si-INHA), and control (si-NC) groups; *n* = 6 donor-derived biological replicates per group. (**C**) Summary of differentially expressed genes (DEGs) between indicated groups. (**D**,**E**) Volcano plots for INHA-OE versus OE-NC (**D**) and si-INHA versus si-NC (**E**). Red, blue, and gray points indicate upregulated DEGs, downregulated DEGs, and genes that did not meet the DEG criteria, respectively. DEGs were defined by FDR < 0.05 and |log2FC| > 1. (**F**,**G**) Heatmaps showing hierarchical clustering of DEGs in INHA-OE versus OE-NC (**F**) and si-INHA versus si-NC (**G**) comparisons.

To examine transcriptional changes associated with altered *INHA* expression in yak Sertoli cells, RNA sequencing was conducted after *INHA* overexpression and knockdown. Principal component analysis (PCA) demonstrated clear separation between the INHA-OE and OE-NC groups, with PC1 accounting for 41.7% of the total variance (Figure 2A). A similar separation was found between the si-INHA and si-NC groups, with PC1 and PC2 accounting for 53.6% and 11.9% of the variance, respectively (Figure 2B). Biological replicates clustered within their respective groups, indicating consistent transcriptomic profiles within each condition.

Using a donor-adjusted differential-expression model and the prespecified criteria of FDR < 0.05 and |log2FC| > 1, 949 DEGs were identified in the INHA-OE group compared to the OE-NC group, including 351 upregulated and 598 downregulated genes (Figure 2C,D). In contrast, comparison of the si-INHA and si-NC groups identified 28 DEGs, including 10 upregulated and 18 downregulated genes (Figure 2C,E). These findings showed that more genes met the differential-expression criteria after *INHA* overexpression than after knockdown under the experimental conditions used. Complete differential-expression results and sample-level transcriptomic data are provided in Supplementary Table S3.

Hierarchical clustering further showed distinct DEG expression patterns between the experimental and corresponding control groups. The heatmaps illustrated the expression patterns of the 949 overexpression-associated DEGs and 28 knockdown-associated DEGs across individual samples (Figure 2F,G). Overall, these results indicate that changes in *INHA* expression are associated with altered transcriptomic profiles in primary yak Sertoli cells.

### 3.3. Functional Enrichment Analysis and GSEA Reveal INHA-Associated Biological Processes and Signaling Pathways

**Figure 3:**
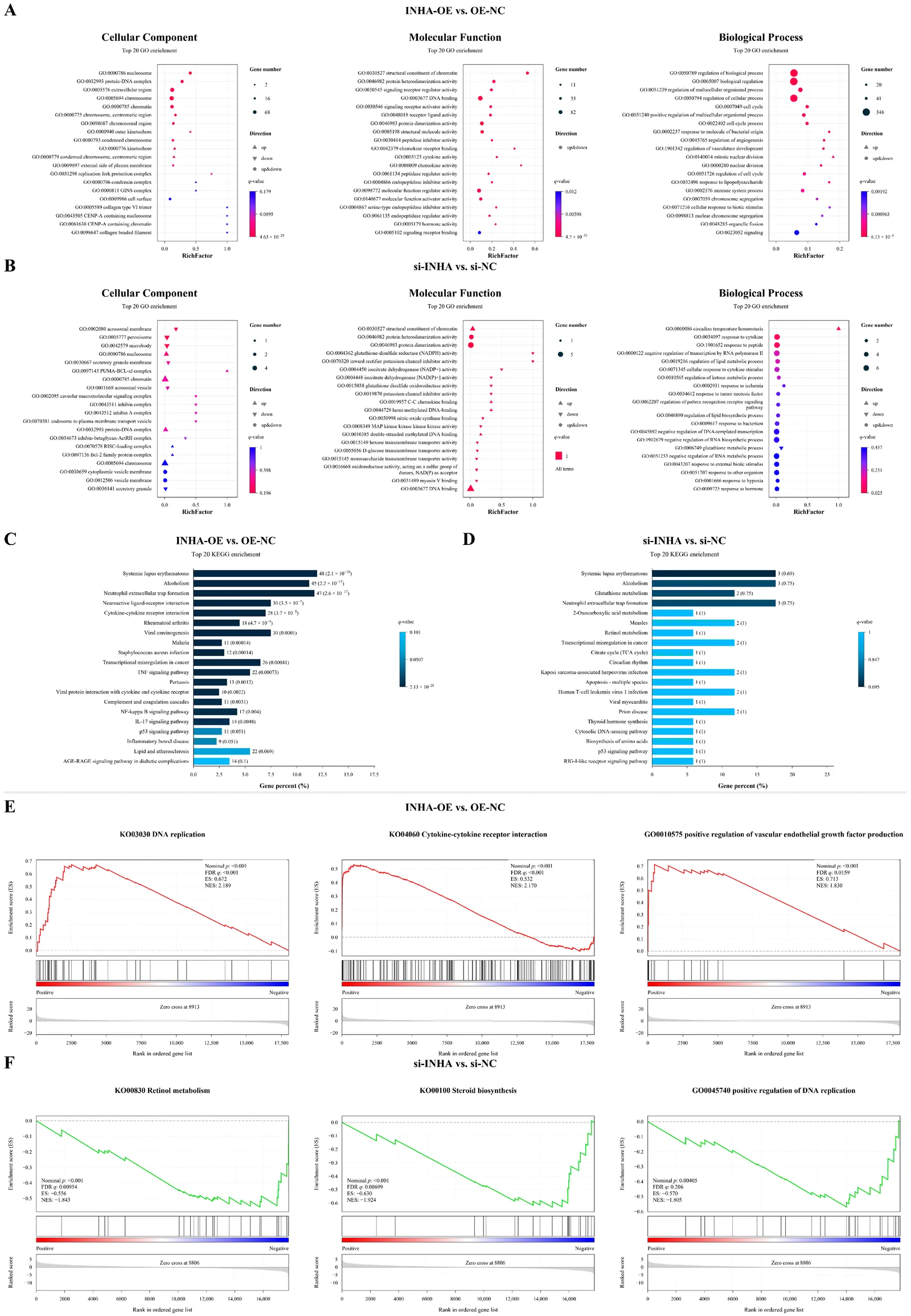
Functional enrichment analysis and GSEA following *INHA* manipulation in yak Sertoli cells. (**A**,**B**) Up to 20 top-ranked GO terms per category in the INHA-OE versus OE-NC (**A**) and si-INHA versus si-NC (**B**) comparisons. The three plots show Cellular Component, Molecular Function, and Biological Process terms. Symbol size represents the number of DEGs, color indicates the *q*-value, and shape indicates upregulated, downregulated, or mixed expression changes. Displayed GO rankings include non-significant terms. (**C**,**D**) Top 20 KEGG pathways in the INHA-OE versus OE-NC (**C**) and si-INHA versus si-NC (**D**) comparisons. Sixteen pathways in (**C**) and none in (**D**) reached *q* < 0.05. (**E**) GSEA plots of selected positively enriched gene sets following *INHA* overexpression; all three displayed gene sets reached FDR *q* < 0.05. (**F**) GSEA plots of selected negatively enriched gene sets following *INHA* knockdown; positive regulation of DNA replication met the preset GSEA threshold but not FDR *q* < 0.05.

GO enrichment analysis showed distinct functional patterns associated with *INHA* overexpression and knockdown. The INHA-OE versus OE-NC comparison revealed that differentially expressed genes (DEGs) were significantly enriched across all three GO domains. Enriched Cellular Component (CC) terms included nucleosome, protein–DNA complex, extracellular region, chromosome, and chromatin. Significant Molecular Function (MF) terms included structural constituent of chromatin, protein heterodimerization activity, DNA binding, and signaling receptor regulator activity. Biological Process (BP) terms included cell cycle, cell cycle process, regulation of cell cycle, regulation of angiogenesis, and regulation of vasculature development (Figure 3A). The GO enrichment profile after *INHA* knockdown was relatively narrow, with only the BP term circadian temperature homeostasis reaching *q* < 0.05 (*q* = 0.025). No CC or MF terms reached this threshold (Figure 3B).

KEGG enrichment analysis showed 16 significantly enriched pathways in the INHA-OE versus OE-NC comparison at *q* < 0.05. These included cytokine–cytokine receptor interaction, TNF signaling, IL-17 signaling, and NF-κB signaling (Figure 3C). In contrast, no KEGG pathways reached the *q* < 0.05 significance threshold in the si-INHA versus si-NC comparison. Displayed pathways, including glutathione metabolism and retinol metabolism, did not meet the significance threshold (Figure 3D).

GSEA was then used to evaluate coordinated expression changes across the transcriptome without restricting the analysis to DEGs. In the INHA-OE versus OE-NC comparison, DNA replication (NES = 2.189; FDR *q* < 0.001), cytokine–cytokine receptor interaction (NES = 2.170; FDR *q* < 0.001), and positive regulation of vascular endothelial growth factor production (NES = 1.830; FDR *q* = 0.016) showed positive enrichment, with all three meeting FDR *q* < 0.05 (Figure 3E). After *INHA* knockdown, retinol metabolism (NES = −1.843; FDR *q* = 0.0095) and steroid biosynthesis (NES = −1.924; FDR *q* = 0.0070) showed significant negative enrichment. Positive regulation of DNA replication was also negatively enriched (NES = −1.805; nominal *p* = 0.0040; FDR *q* = 0.206), meeting the preset GSEA threshold but not the more stringent FDR *q* < 0.05 criterion (Figure 3F).

### 3.4. DIA-Based Quantitative Proteomics Reveals Global Protein Abundance Changes Following INHA Manipulation

**Figure 4:**
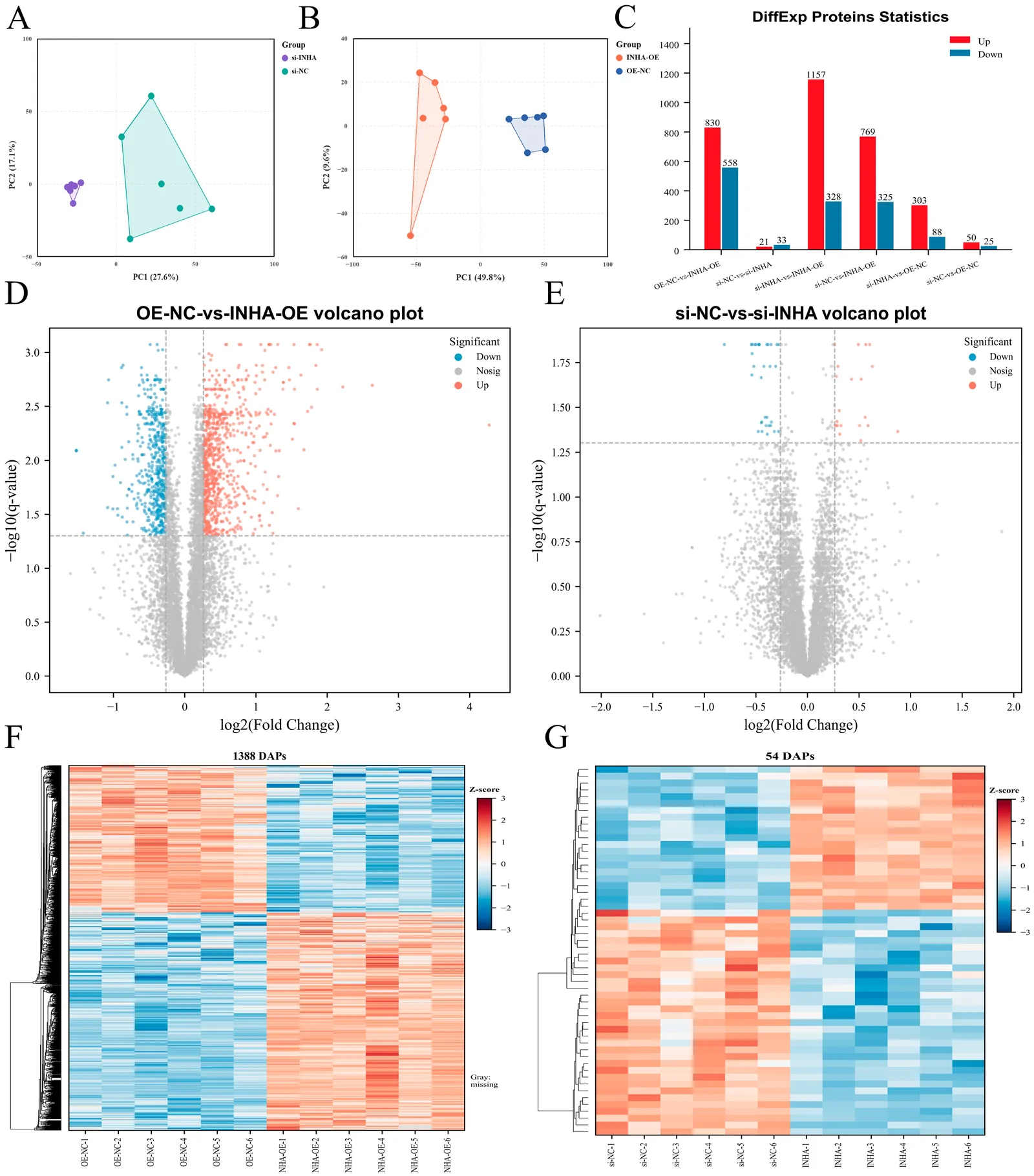
DIA-based quantitative proteomic profiling of yak Sertoli cells following *INHA* manipulation. (**A**,**B**) Principal component analysis of the si-INHA and si-NC groups (**A**) and the INHA-OE and OE-NC groups (**B**); *n* = 6 donor-derived biological replicates per group. The percentages shown on the axes indicate the proportions of total variance explained by the corresponding principal components. (**C**) Numbers of upregulated and downregulated proteins in the indicated pairwise comparisons. (**D**,**E**) Volcano plots of differentially abundant proteins in INHA-OE versus OE-NC (**D**) and si-INHA versus si-NC (**E**). Red, blue, and gray points indicate upregulated DAPs, downregulated DAPs, and proteins that did not meet the DAP criteria, respectively. Differentially abundant proteins (DAPs) were defined by donor-paired tests with BH-adjusted FDR < 0.05 and (FC > 1.2 or FC < 1/1.2). Positive log2FC indicates higher abundance in the treatment group than in its corresponding control. (**F**,**G**) Heatmaps of DAPs in the overexpression (**F**) and knockdown (**G**) comparisons. Colors represent row-wise Z-scores of log2-transformed protein abundance; gray indicates missing values.

To further evaluate proteomic changes associated with altered *INHA* expression, DIA-based quantitative proteomic analysis was conducted in yak Sertoli cells. A total of 94,011 precursors, 85,470 peptides, 7345 protein groups, and 7493 proteins were identified. PCA revealed a clear separation between the si-INHA and si-NC groups, with PC1 and PC2 accounting for 27.6% and 17.1% of the total variance, respectively (Figure 4A). The INHA-OE and OE-NC groups similarly showed distinct clustering, with PC1 and PC2 accounting for 49.8% and 9.6% of the variance, respectively (Figure 4B). Although some biological replicates showed a certain degree of dispersion, the treatment groups and their corresponding control groups still exhibited distinct overall protein-abundance profiles.

Using donor-paired tests and the screening criteria of Benjamini–Hochberg-adjusted FDR < 0.05 and (FC > 1.2 or FC < 1/1.2), 1388 differentially abundant proteins (DAPs) were identified in the INHA-OE versus OE-NC comparison, including 830 upregulated and 558 downregulated proteins. In the si-INHA versus si-NC comparison, 54 DAPs were identified, of which 21 were upregulated and 33 were downregulated (Figure 4C). Therefore, under the applied screening criteria, more proteins met the differential-abundance criteria after *INHA* overexpression than after knockdown. Complete differential-abundance results, sample-level protein abundance matrices, and donor metadata are provided in Supplementary Table S4.

Volcano plots further illustrate bidirectional changes in protein abundance in both comparisons (Figure 4D,E). Heatmaps with hierarchical clustering showed the abundance patterns of the 1388 overexpression-associated DAPs and 54 knockdown-associated DAPs across individual samples (Figure 4F,G). Overall, these results indicate that perturbation of *INHA* expression is associated with altered protein-abundance profiles in primary yak Sertoli cells.

### 3.5. Proteomic Functional Enrichment and GSEA Identify INHA-Associated Molecular Pathways

**Figure 5:**
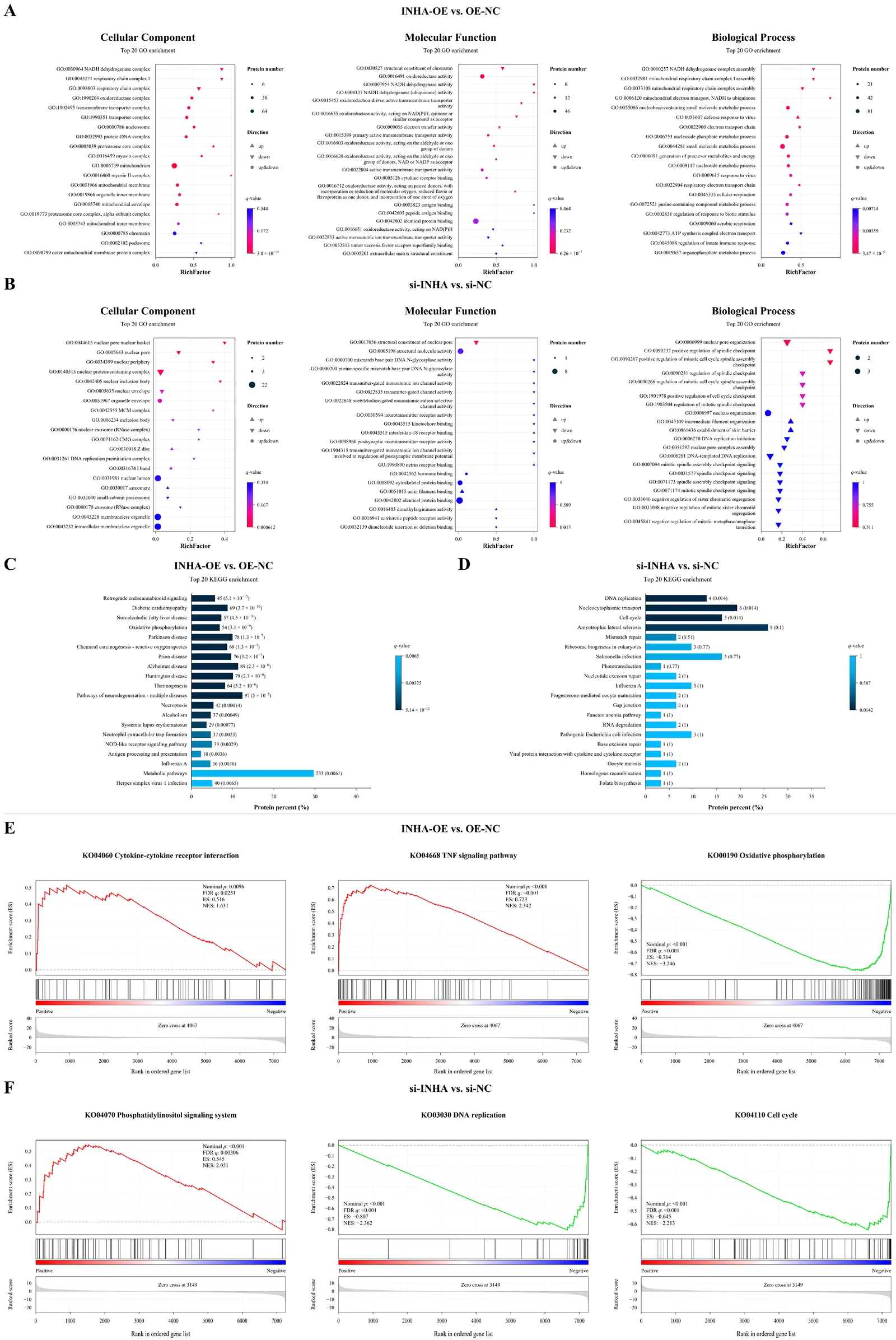
Proteomic functional enrichment and GSEA following *INHA* manipulation in yak Sertoli cells. (**A**,**B**) Up to 20 top-ranked GO terms per category in INHA-OE versus OE-NC (**A**) and si-INHA versus si-NC (**B**). Point position, size, color, and shape represent RichFactor, DAP number, *q*-value, and upregulated, downregulated, or mixed abundance changes, respectively. (**C**,**D**) Top 20 KEGG pathways in the corresponding comparisons; bar length and color indicate protein percentage and *q*-value. Displayed GO and KEGG rankings include non-significant terms. (**E**,**F**) GSEA of selected pathways in the overexpression (**E**) and knockdown (**F**) comparisons; all shown gene sets reached FDR *q* < 0.05. Positive and negative NES indicate enrichment toward higher and lower protein abundance in the treatment group relative to its control, respectively. NES, normalized enrichment score; FDR, false discovery rate.

To characterize functional patterns associated with *INHA* perturbation, GO and KEGG enrichment analyses were performed using DAPs. In the INHA-OE versus OE-NC comparison, enriched Cellular Component terms included NADH dehydrogenase complex, respiratory chain complex I, respiratory chain complex, and oxidoreductase complex. Molecular Function terms mainly involved NADH dehydrogenase activity, oxidoreductase activity, and structural constituent of chromatin, whereas enriched Biological Processes included mitochondrial respiratory chain assembly, electron transport, and generation of precursor metabolites and energy (Figure 5A). In contrast, significantly enriched Cellular Component terms in the si-INHA versus si-NC comparison included nuclear pore nuclear basket, nuclear pore, nuclear periphery, nuclear protein-containing complex, and nuclear inclusion body. Structural constituent of nuclear pore was the only significant Molecular Function term, and no Biological Process terms reached *q* < 0.05 (Figure 5B).

KEGG enrichment analysis further revealed distinct pathway profiles associated with *INHA* overexpression and knockdown. DAPs in the INHA-OE versus OE-NC comparison were significantly enriched in oxidative phosphorylation, thermogenesis, NOD-like receptor signaling, and necroptosis (Figure 5C). In the si-INHA versus si-NC comparison, only DNA replication, nucleocytoplasmic transport, and cell cycle reached *q* < 0.05 (Figure 5D).

GSEA was then performed using proteins with valid differential-abundance results to evaluate coordinated changes without restricting the analysis to proteins meeting the differential-abundance criteria. In the INHA-OE versus OE-NC comparison, cytokine–cytokine receptor interaction (NES = 1.631; FDR *q* = 0.025) and TNF signaling pathway (NES = 2.342; FDR *q* < 0.001) showed positive enrichment, whereas oxidative phosphorylation was negatively enriched (NES = −3.246; FDR *q* < 0.001) (Figure 5E). In the si-INHA versus si-NC comparison, the phosphatidylinositol signaling system was positively enriched (NES = 2.051; FDR *q* = 0.0031), whereas DNA replication (NES = −2.362; FDR *q* < 0.001) and cell cycle (NES = −2.213; FDR *q* < 0.001) were negatively enriched (Figure 5F). Overall, these results indicate that *INHA* perturbation is associated with protein-abundance changes involving cytokine-related signaling, mitochondrial respiratory processes, nuclear pore-associated structures, and DNA replication and cell-cycle pathways.

### 3.6. Integrated Transcriptomic and Proteomic Analysis Identifies INHA-Responsive Molecular Signatures

**Figure 6:**
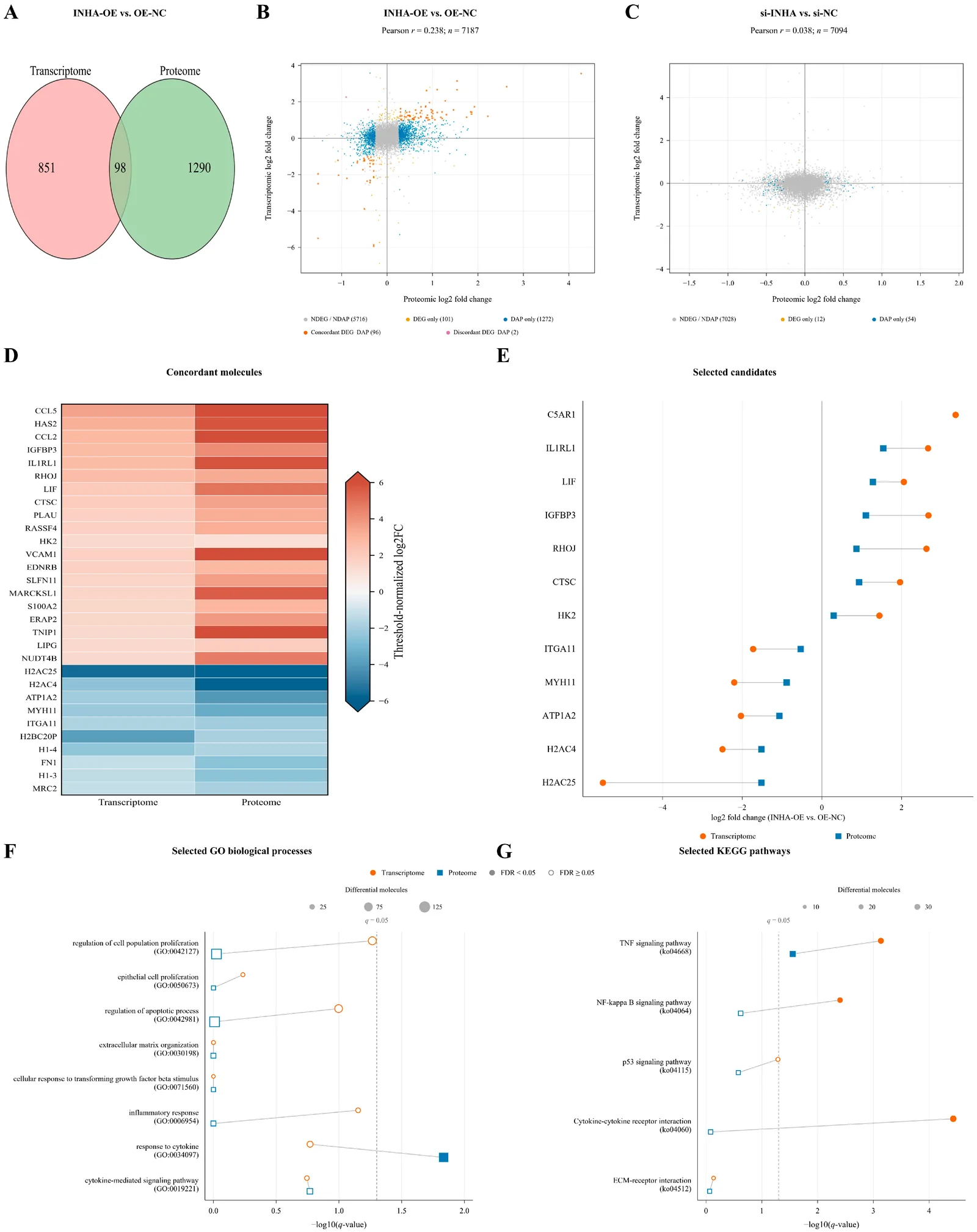
Integrated transcriptomic and proteomic analysis of *INHA*-responsive molecular changes. (**A**) Overlap between DEGs and DAPs in INHA-OE versus OE-NC. (**B**,**C**) Correlations between transcriptomic and proteomic log2 fold changes across matched molecules with valid statistical results. (**D**) Heatmap of 30 selected concordantly altered molecules following *INHA* overexpression. Values represent log2 fold changes scaled to the respective differential-change thresholds. (**E**) Paired mRNA–protein log2 fold changes of selected candidates, not restricted to the significant overlap; unavailable protein effects are indicated. (**F**,**G**) Paired enrichment plots of selected GO Biological Process terms (**F**) and KEGG pathways (**G**). Orange circles and blue squares represent transcriptomic and proteomic results, respectively. Filled and open symbols indicate FDR < 0.05 and FDR ≥ 0.05, respectively. Symbol size represents the number of differential molecules, and gray lines connect the same terms.

To assess the relationship between transcript- and protein-level responses to altered *INHA* expression, transcriptomic and proteomic data were analyzed jointly. In the comparison between INHA-OE and OE-NC, a total of 98 molecules met the differential criteria at both the mRNA and protein levels. Of these, 96 mRNA–protein pairs showed concordant changes across the two datasets, whereas two showed discordant changes (Figure 6A). In contrast, no molecules met the differential criteria in both datasets in the si-INHA versus si-NC comparison. Complete matched mRNA–protein results and the ranked list of concordant overexpression candidates are provided in Supplementary Table S5.

Across the 7187 matched mRNA–protein pairs with valid differential-expression results in both datasets, log2 fold changes after *INHA* overexpression showed a weak positive correlation between the transcriptomic and proteomic datasets (Pearson’s *r* = 0.238; Figure 6B). After *INHA* knockdown, the correlation across 7094 matched pairs was close to zero (*r* = 0.038; Figure 6C). These coefficients were used to describe the overall correspondence between transcript- and protein-level changes. Given the presence of concordantly altered molecules in the overexpression comparison, subsequent candidate-level analyses focused on INHA-OE versus OE-NC.

Among the concordantly altered molecules selected for visualization after *INHA* overexpression, *CCL5*, *CCL2*, *IGFBP3*, *IL1RL1*, *LIF*, *CTSC*, and *HK2* were upregulated at both the mRNA and protein levels, whereas *ATP1A2*, *MYH11*, *ITGA11*, and *FN1* were downregulated at both levels (Figure 6D). Paired visualization of selected candidates similarly showed concordant upregulation of *IL1RL1*, *LIF*, *IGFBP3*, *RHOJ*, *CTSC*, and *HK2* and concordant downregulation of *ITGA11*, *MYH11*, *ATP1A2*, *H2AC4*, and *H2AC25*. *C5AR1* was shown at the transcript level only because its protein fold-change estimate was unavailable under the minimum complete-pair criterion (Figure 6E).

Cross-omics comparison of the selected GO terms showed that “response to cytokine” was significantly enriched in the proteomic analysis but not in the transcriptomic analysis. The other selected terms, including “cytokine-mediated signaling pathway”, regulation of cell population proliferation, epithelial cell proliferation, regulation of apoptotic process, extracellular matrix organization, inflammatory response, and cellular response to transforming growth factor beta stimulus, did not reach the FDR-adjusted *q* < 0.05 threshold in either dataset (Figure 6F).

KEGG enrichment analysis revealed that the “TNF signaling pathway” was significantly enriched at both omics levels. The “NF-κB signaling pathway” and “cytokine–cytokine receptor interaction” reached statistical significance only in the transcriptomic analysis, whereas “ECM–receptor interaction” and the “p53 signaling pathway” did not meet the FDR-adjusted *q* < 0.05 threshold in either dataset (Figure 6G). Overall, these results highlight TNF signaling as a shared enrichment finding and identify concordantly altered molecules as candidate *INHA*-responsive molecular signatures.

### 3.7. Targeted qRT-PCR Confirmation of Selected INHA-Responsive Genes

**Figure 7:**
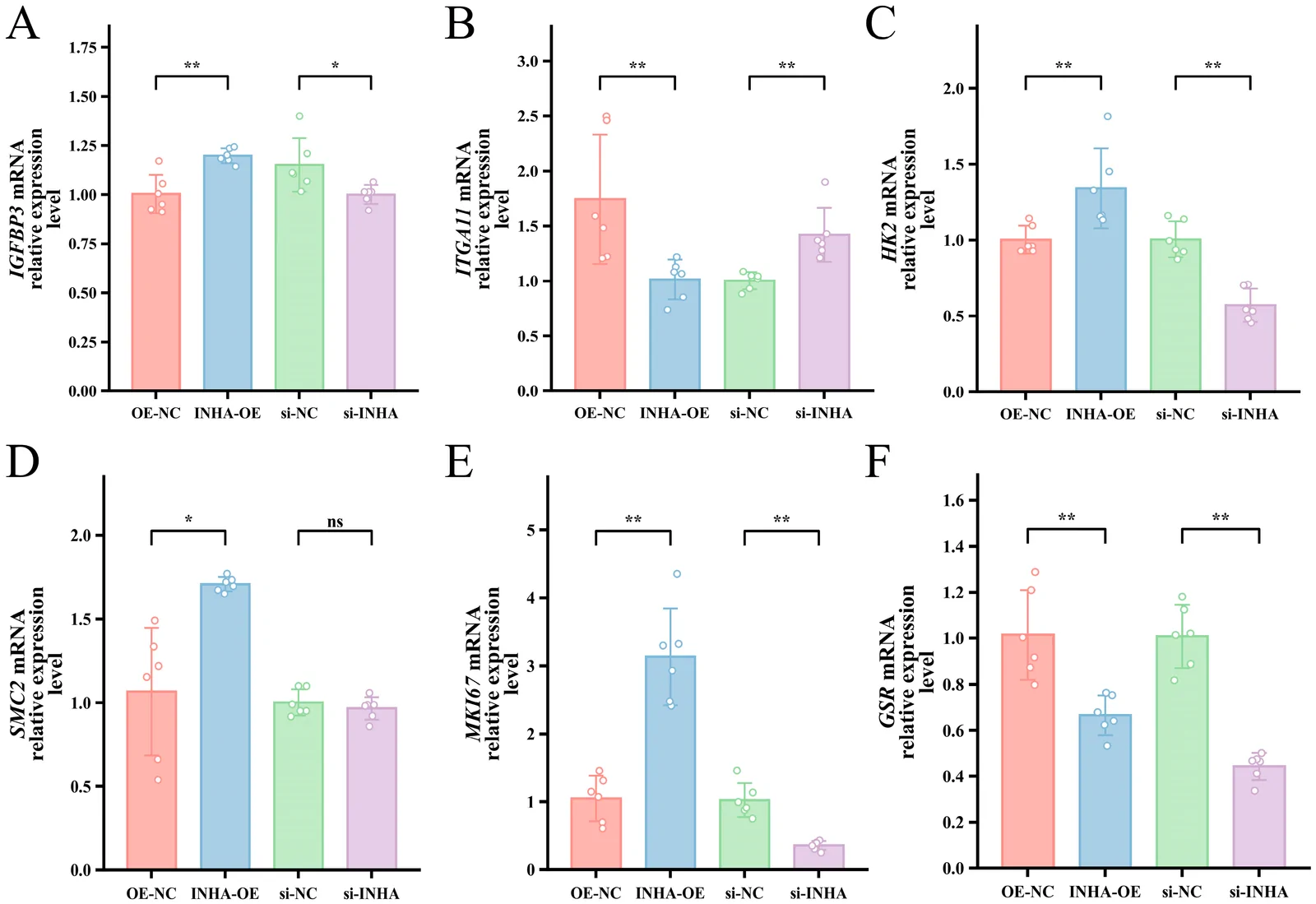
qRT-PCR analysis of selected *INHA*-responsive genes in yak Sertoli cells. Relative mRNA levels of *IGFBP3* (**A**), *ITGA11* (**B**), *HK2* (**C**), *SMC2* (**D**), *MKI67* (**E**), and *GSR* (**F**) were normalized to *ACTB* (β-actin). Data are mean ± SD (n = 6 donor-derived biological replicates per group); dots represent individual replicates. Each treatment was compared with its corresponding negative control using two-tailed unpaired Student’s *t*-tests. ns, not significant; * *p* < 0.05; ** *p* < 0.01.

To further assess selected transcript-level responses to *INHA* manipulation, the mRNA levels of *IGFBP3*, *ITGA11*, *HK2*, *SMC2*, *MKI67*, and *GSR* were evaluated using qRT-PCR, with six biological replicates per group. Compared with the OE-NC group, *INHA* overexpression significantly upregulated the expression of *IGFBP3*, *HK2*, *SMC2*, and *MKI67*, whereas it downregulated *ITGA11* and *GSR* (Figure 7A–F). *INHA* knockdown significantly reduced the expression of *IGFBP3*, *HK2*, *MKI67*, and *GSR* and increased the expression of *ITGA11*. No significant difference in *SMC2* expression was detected between the si-INHA and si-NC groups.

Notably, *ITGA11* exhibited reciprocal expression patterns under the two experimental conditions, with reduced expression after *INHA* overexpression and elevated expression after *INHA* knockdown. Overall, the qRT-PCR results were consistent with the direction of RNA-Seq changes for *IGFBP3*, *ITGA11*, *HK2*, *SMC2*, and *MKI67* in the overexpression comparison and for *GSR* in the knockdown comparison. These observations provide targeted transcript-level support for selected *INHA*-responsive expression changes.

### 3.8. CCK-8 Signal, Apoptosis, and Microtubule Organization Following INHA Manipulation in Yak Sertoli Cells

**Figure 8:**
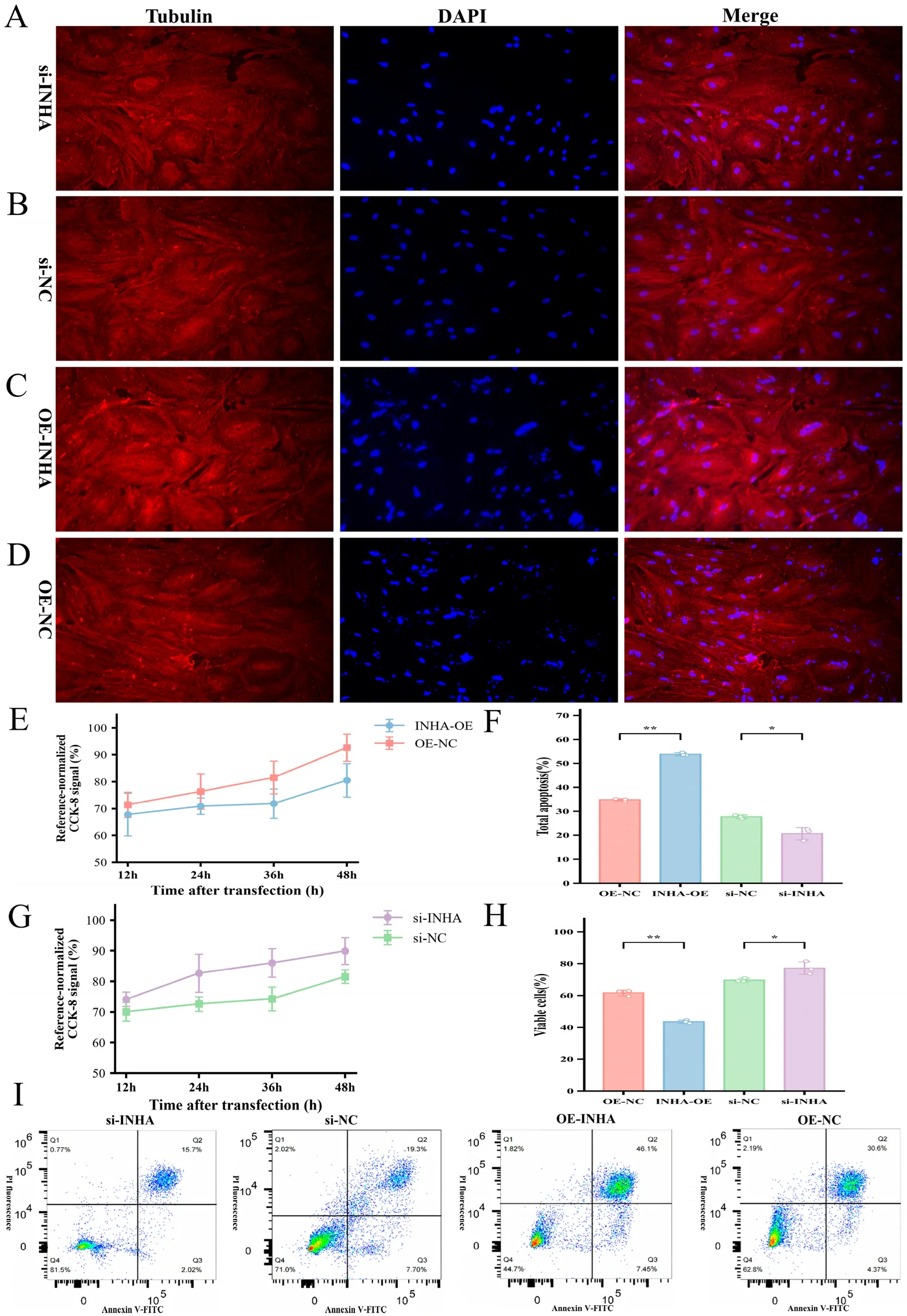
CCK-8 signal, apoptosis, and β-tubulin organization following *INHA* manipulation in yak Sertoli cells. (**A**–**D**) Representative β-tubulin immunofluorescence images in the si-INHA (**A**), si-NC (**B**), INHA-OE (**C**), and OE-NC (**D**) groups. β-Tubulin and nuclei are shown in red and blue, respectively. (**E**,**G**) Reference-normalized CCK-8 signal measured at 12, 24, 36, and 48 h following overexpression (**E**) or knockdown (**G**); six technical replicate wells from a single pooled cell preparation per group at each time point. Error bars indicate SD across technical wells; no inferential statistical comparisons were performed for these panels. (**F**,**H**) Total apoptotic-cell (**F**) and viable-cell (**H**) proportions; *n* = 3 independent donor-derived biological replicates per group. In panels (**E**–**H**), pink, blue, green, and purple represent the OE-NC, INHA-OE, si-NC, and si-INHA groups, respectively. (**I**) Representative Annexin V-FITC/PI plots. Q1 denotes Annexin V^−^/PI^+^ cells; Q2, Q3, and Q4 denote late apoptotic, early apoptotic, and viable cells, respectively. Total apoptosis = Q2 + Q3. Data are mean ± SD. For (**F**,**H**), treatments were compared with their corresponding negative controls using two-tailed unpaired Student’s *t*-tests. * *p* < 0.05; ** *p* < 0.01.

To characterize cellular responses associated with altered *INHA* expression in yak Sertoli cells, β-tubulin immunofluorescence, the CCK-8 assay, and Annexin V-FITC/PI flow cytometry were used to examine microtubule organization, CCK-8 signal, and apoptosis, respectively. Cytoplasmic β-tubulin-positive filamentous structures were observed in all four groups (Figure 8A–D). Compared with the si-NC group, β-tubulin-positive structures in the si-INHA group appeared more interlaced in some regions. Differences in the local distribution and orientation of β-tubulin-positive structures were also observed between the INHA-OE and OE-NC groups. These observations describe qualitative differences in β-tubulin staining patterns associated with *INHA* perturbation.

Within the single pooled cell preparation, the mean reference-normalized CCK-8 signal increased from 12 to 48 h in all four groups (Figure 8E,G). The INHA-OE group showed lower mean values than the OE-NC group at all measured time points, whereas the si-INHA group showed higher mean values than the si-NC group. Values are presented as mean ± SD across six technical replicate wells per group at each time point.

Annexin V-FITC/PI flow cytometry showed differences in apoptotic and viable-cell proportions (Figure 8F,H,I). Compared with OE-NC, the INHA-OE group had an apoptotic-cell proportion 18.99 percentage points higher (*p* = 1.78 × 10^−6^) and a viable-cell proportion 18.04 percentage points lower (*p* = 0.000102). Compared with si-NC, the si-INHA group had an apoptotic-cell proportion 7.10 percentage points lower (*p* = 0.0103) and a viable-cell proportion 7.59 percentage points higher (*p* = 0.0332). Individual quadrant proportions, mean differences with 95% confidence intervals, and complete statistical results are provided in Supplementary Table S5.

## 4. Discussion

### 4.1. INHA Manipulation Alters the Molecular and Functional State of Yak Sertoli Cells

In this study, we established *INHA* overexpression and knockdown models in primary yak Sertoli cells from 3-month-old donors to investigate the molecular and cellular responses associated with altered *INHA* expression. Integration of RNA-Seq and directDIA quantitative proteomics identified more DEGs and DAPs following overexpression than knockdown in the respective treatment–control comparisons. These changes involved several biological processes relevant to Sertoli-cell function, including cytokine-related signaling, DNA replication, cell-cycle regulation, metabolic processes, and cell–matrix interactions. Integrated analysis identified concordantly altered mRNA–protein candidates, while qRT-PCR provided targeted support for selected transcript-level changes. Overall, these findings show that altering *INHA* expression is associated with diverse molecular changes in yak Sertoli cells; however, the responses to overexpression and knockdown were not uniformly reciprocal. Differences in perturbation magnitude, nonlinear cellular responses, and the different perturbation methods and negative controls may contribute to this asymmetry. DEG and DAP counts therefore describe the respective analyses rather than directly comparable biological effect sizes.

Although *INHA* mRNA increased markedly, the mean INHA/β-actin band-intensity ratio was 1.85-fold that of the OE-NC group, indicating different magnitudes of change at the transcript and protein levels. These findings characterize *INHA*-associated responses under the tested conditions, although some cytokine-related, metabolic, and apoptotic changes may partly reflect expression-associated cellular stress. Further assessment at lower expression levels would help clarify the specificity and physiological relevance of these responses.

Sertoli cells are the primary source of inhibin B in the male reproductive system [29]. Mature inhibin B is produced by association of the inhibin α-subunit (encoded by *INHA*) with the βB subunit (encoded by *INHBB*) and contributes to the negative-feedback regulation of pituitary FSH secretion. Circulating inhibin B levels are therefore closely associated with Sertoli cell function and spermatogenic activity [14]. Evidence from animal models also suggests a broader role for the inhibin α-subunit in gonadal homeostasis. In particular, *Inha*-deficient mice exhibit abnormal gonadal cell proliferation and gonadal tumors, indicating the importance of the inhibin α-subunit in maintaining gonadal homeostasis [30]. Our findings extend the characterization of *INHA*-associated responses to primary yak Sertoli-cell cultures, where altered *INHA* expression was associated with transcriptomic and proteomic responses, differences in flow-cytometric apoptosis measurements, and qualitative differences in β-tubulin staining patterns. These findings concern *INHA* expression and α-subunit abundance, rather than directly demonstrating changes in mature inhibin production or activity. These observations provide a basis for further exploration of how *INHA* and the inhibin-related regulatory system contribute to Sertoli-cell function in yak.

### 4.2. INHA Is Associated with Cell Survival and Cell-Cycle-Related Molecular Programs

Changes in DNA replication and cell-cycle-related molecular programs were a prominent feature following *INHA* perturbation. Transcriptomic analysis revealed that genes differentially expressed following *INHA* overexpression were enriched in chromatin organization and cell-cycle-related functions; GSEA also showed positive enrichment of the DNA replication gene set. At the proteomic level, *INHA* knockdown was associated with negative enrichment of the DNA replication and cell cycle gene sets. Consistent with the transcriptomic findings, qRT-PCR detected increased expression of *MKI67* and *SMC2* following *INHA* overexpression. SMC2 is a component of condensin complexes involved in chromosome organization [31]. These findings suggest that altered *INHA* expression is associated with changes in molecular programs involved in DNA replication and cell-cycle regulation.

The CCK-8 assay provided descriptive observations from a single pooled cell preparation. Mean reference-normalized CCK-8 values across six technical replicate wells were generally lower after *INHA* overexpression and higher after *INHA* knockdown relative to their respective controls. Since the CCK-8 signal depends on both the number of live cells and cellular metabolic activity, these measurements do not distinguish changes in viable-cell number from changes in metabolic activity and do not directly measure cell proliferation [32,33]. Without independent biological replication, the observed differences cannot establish reproducible treatment effects across donor-derived cultures. Evidence for differences in cell survival was provided by Annexin V-FITC/PI flow cytometry, which distinguishes viable and apoptotic cell populations [34]. Compared with their respective negative controls, the INHA-OE group showed a higher proportion of apoptotic cells and a lower proportion of viable cells, whereas the si-INHA group showed the opposite pattern. Together, the flow-cytometry findings associate *INHA* perturbation with changes in Sertoli-cell survival and apoptosis. The observed changes in *MKI67*, *SMC2*, DNA replication, and cell-cycle-related processes are therefore best interpreted as molecular responses associated with cell-cycle regulation rather than direct evidence of altered cell proliferation.

### 4.3. Cytokine Signaling Is a Prominent INHA-Responsive Feature

Cytokine-related signaling emerged as a prominent functional feature across the transcriptomic and proteomic datasets. GO analysis revealed that “response to cytokine” was significantly enriched at the protein level, whereas KEGG analysis identified significant enrichment of the TNF signaling pathway in both datasets and the NF-κB signaling pathway at the transcriptomic level. GSEA further showed positive enrichment of cytokine–cytokine receptor interaction in both datasets following *INHA* overexpression. Immune- and cytokine-related molecules, such as *CCL2*, *CCL5*, *IL1RL1*, *LIF*, and *CTSC*, also showed concordant increases at the mRNA and protein levels after *INHA* overexpression. Overall, these findings suggest that altered *INHA* expression, particularly overexpression, is associated with coordinated changes in cytokine- and immune-related molecular programs in yak Sertoli cells.

Sertoli cells are not merely structural support cells, but also key regulators of the testicular immune microenvironment [35]. They can modulate local immune responses through cytokines, chemokines, and other immunoregulatory factors, and play a role in maintaining local immune homeostasis and providing an environment compatible with germ-cell development [36]. Previous studies have shown that TNF-α activates NF-κB signaling in Sertoli cells and can regulate the expression of germ-cell-supporting factors, including GDNF [37,38]. TNF signaling has also been implicated in the dynamic regulation of the blood–testis barrier [39]. Therefore, the cytokine-related enrichment observed after *INHA* perturbation may reflect changes in the immunoregulatory and stress-response state of Sertoli cells, providing a biological context for the accompanying changes in cell survival.

Among the concordantly altered candidates, *CCL2*, *CCL5*, *IL1RL1*, *LIF*, and *CTSC* represent molecules of interest for assessing the relationship between *INHA* expression and cytokine-related responses [40]. Their coordinated changes, together with the apoptosis findings, identify cytokine-related molecular programs as potential contributors to the cellular response to *INHA* perturbation. However, enrichment does not itself establish TNF/NF-κB pathway activation or a causal link to apoptosis [39,41]. Therefore, future studies involving the direct assessment of pathway activity and targeted manipulation of individual signaling components would clarify whether TNF/NF-κB signaling has a mechanistic role in the observed phenotype.

### 4.4. INHA-Related Metabolic and Structural Responses

Proteomic GSEA revealed that oxidative phosphorylation was negatively enriched following *INHA* overexpression, whereas both *HK2* mRNA levels and HK2 protein abundance increased, with the transcript-level changes further supported by qRT-PCR. These results suggest coordinated changes in molecular programs related to mitochondrial energy metabolism and glucose utilization. Sertoli cells have specialized metabolic functions and can metabolize glucose to produce lactate, thereby providing metabolic support to developing germ cells. Previous research has demonstrated hypoxia-associated glucose metabolic reprogramming in yak Sertoli cells [21], while Sertoli cell-specific *Ldha* deletion reduces lactate production and impairs spermatogenesis in mice [42]. Therefore, the present findings highlight metabolism-related molecular responses associated with *INHA* perturbation, rather than directly demonstrating altered metabolic flux. Direct assessment of glycolytic activity, mitochondrial respiration, and lactate production would help establish the functional consequences of these molecular changes.

Transcriptomic and integrated analyses also revealed changes in molecules associated with the extracellular matrix and cell–matrix interactions. *FN1* and *ITGA11* showed concordant decreases at both the mRNA and protein levels after *INHA* overexpression, highlighting cell–matrix-associated molecules as part of the response to altered *INHA* expression. In parallel, β-tubulin immunofluorescence images suggested qualitative differences in the distribution and arrangement of β-tubulin-positive structures after *INHA* perturbation. Microtubules are involved in maintaining Sertoli cell morphology and intracellular transport and contribute to the coordinated movement of developing germ cells during spermiogenesis [43,44]. Overall, these results associate altered *INHA* expression with metabolism- and cell–matrix-related molecular changes, accompanied by qualitative differences in microtubule organization.

### 4.5. Integrated Analysis Prioritizes Candidate INHA-Responsive Molecular Signatures

Integrated transcriptomic–proteomic analysis was used to assess the relationship between mRNA- and protein-level changes and altered *INHA* expression. After *INHA* overexpression, 98 molecules met the differential criteria in both datasets, of which 96 exhibited concordant changes at the transcript and protein levels. The correlation between transcriptomic and proteomic log2 fold changes across matched molecules with valid statistical results was weakly positive (Pearson’s *r* = 0.238). These results describe complementary aspects of the cross-omics response: the correlation summarizes the overall relationship between mRNA- and protein-level changes, whereas the overlap identifies molecules meeting the differential criteria in both datasets. The relatively modest overall correlation may reflect post-transcriptional regulation, protein degradation, and differences in the timing of transcript- and protein-level responses [20,45]. Because no molecules met the differential criteria in both datasets following *INHA* knockdown, subsequent cross-omics interpretation focused primarily on the overexpression condition.

Molecules meeting the differential criteria in both datasets included candidates associated with cytokine-related signaling, metabolic regulation, and cell–matrix interactions. CCL2, CCL5, IL1RL1, LIF, and CTSC were associated with cytokine-related processes, whereas HK2 was linked to glucose metabolism. FN1 and ITGA11 are involved in extracellular matrix and cell adhesion-related functions. qRT-PCR results supported the direction of RNA-Seq changes for *IGFBP3*, *ITGA11*, *HK2*, *SMC2*, and *MKI67* in the overexpression comparison and for *GSR* in the knockdown comparison. Integration of transcriptomic and proteomic evidence therefore provides a useful basis for prioritizing selected *INHA*-responsive molecules for subsequent functional exploration.

The molecular findings of the current study can be considered alongside the cellular phenotypes observed following *INHA* perturbation. Changes in cytokine-, metabolism-, cell–matrix-, and cell-cycle-related molecular programs occurred together with differences in Annexin V-FITC/PI-defined apoptosis and qualitative β-tubulin staining patterns. Although these observations do not establish direct mechanistic links between individual candidate molecules and the observed cellular phenotypes, they provide biologically relevant directions for future investigations. *CCL2*, *CCL5*, *IL1RL1*, *LIF*, *CTSC*, *HK2*, *FN1*, and *ITGA11* can serve as candidate molecules for targeted studies to investigate their potential contributions to *INHA*-associated Sertoli-cell responses.

### 4.6. Study Scope and Future Directions

The results of this study should be interpreted within the scope of experimental design. The primary cultures were derived from 3-month-old yaks, and the findings therefore concern an immature, in vitro Sertoli-cell model rather than adult testicular function. The enrichment procedure, cell morphology, and GATA4/vimentin staining supported the use of these cultures as a yak Sertoli-cell model. However, possible contributions from other testicular cell types should be considered when interpreting the transcriptomic and proteomic findings. The correspondence between the overexpression level and physiological *INHA* expression has not been established. Key molecular and cellular responses were not examined across lower expression levels or against an expression-matched unrelated-protein control; therefore, a contribution from expression-associated stress cannot be excluded.

Although *INHA* knockdown was confirmed at both the mRNA and protein levels, downstream phenotypes were evaluated using only si-INHA-1062. Without confirmation using a second independent siRNA or a rescue experiment, sequence-specific off-target contributions to these phenotypes cannot be excluded. CCK-8 measurements were limited to technical replicates from a single pooled cell preparation and therefore describe within-preparation observations without establishing between-donor reproducibility. Future studies using independent donor-derived preparations and an appropriate treatment × time model are needed to evaluate treatment effects over time. In addition, the scaledTPM-derived RNA-Seq input warrants further verification against the original quantification outputs.

*INHA* encodes the α-subunit common to both inhibin A and inhibin B, whereas formation of the mature inhibin proteins requires association of the α-subunit with the corresponding β-subunit [46]. Therefore, the changes in *INHA* expression and α-subunit abundance detected in this study should not be interpreted as direct evidence of altered production or secretion of mature inhibin A or inhibin B. Furthermore, functional enrichment and cross-omics consistency analyses identified molecular signatures associated with *INHA* perturbation; however, pathway activation, cytokine secretion, and direct regulatory relationships among candidate molecules still need to be confirmed through targeted functional assays.

Future studies should further examine whether *INHA* perturbation alters the production and secretion of mature inhibin A and inhibin B and assess the functional activity of the pathways highlighted by the omics analyses. Graded *INHA* overexpression, an expression-matched unrelated-protein control, and measurements of cellular stress would help determine whether the identified responses persist at lower expression levels. A second independent *INHA*-targeting siRNA and rescue with an siRNA-resistant *INHA* construct would further assess knockdown specificity. Combining direct measures of DNA synthesis and cell-cycle progression, such as EdU incorporation and cell-cycle distribution, would help elucidate the relationship between the observed cell-cycle-related changes and Sertoli-cell proliferation. Integrating targeted pathway assays with functional manipulation of selected candidate molecules would help define the mechanisms linking *INHA* expression to Sertoli-cell function in yaks.

## 5. Conclusions

Using *INHA* overexpression and knockdown models in primary Sertoli cells from 3-month-old yaks, we characterized associated molecular and cellular responses through RNA-Seq, directDIA quantitative proteomics, and integrated transcriptomic–proteomic analysis. The molecular changes involved cytokine signaling, DNA replication, cell-cycle-related molecular programs, energy metabolism, and cell–matrix-associated molecules. Among these, the TNF signaling pathway showed significant enrichment at both transcriptomic and proteomic levels, whereas the NF-κB signaling pathway was significantly enriched at the transcriptomic level. Integrated analysis further identified a set of concordantly altered mRNA–protein pairs, including *CCL2*, *CCL5*, *IL1RL1*, *LIF*, *CTSC*, *HK2*, *FN1*, and *ITGA11*, as candidate molecules for future functional investigation. Selected RNA-Seq expression patterns were also supported by the qRT-PCR results. At the cellular level, flow cytometry showed that *INHA* overexpression was associated with increased apoptosis and a decreased proportion of viable cells, whereas INHA knockdown produced the opposite pattern. β-Tubulin immunofluorescence also revealed qualitative differences in microtubule staining patterns following *INHA* perturbation. Together, these findings indicate associations between altered *INHA* expression and cytokine- and metabolism-related molecular responses, changes in cell survival, and qualitative differences in microtubule organization, providing a basis for future studies to investigate the role of *INHA* in yak Sertoli-cell biology and its relationship to the broader inhibin-related regulatory system.

## Data Availability

The raw transcriptomic sequencing data generated in this study have been deposited in the Genome Sequence Archive (GSA) at the National Genomics Data Center (NGDC), China National Center for Bioinformation/Beijing Institute of Genomics, Chinese Academy of Sciences, under accession number CRA049611 and are publicly accessible at https://ngdc.cncb.ac.cn/gsa (accessed on 10 September 2026). The proteomic datasets have been deposited in the OMIX database under accession number OMIX020087 and are publicly accessible at https://ngdc.cncb.ac.cn/omix (accessed on 10 September 2026). Processed transcriptomic and proteomic matrices, the derived RNA-Seq analysis-input matrix, omics sample metadata, and complete differential-analysis results are provided in Supplementary Tables S3 and S4. Integrated results, complete candidate rankings, analysis settings, and individual Western blot and flow-cytometry summary data with detailed statistics are provided in Supplementary Table S5. Relative qRT-PCR expression data for selected genes are provided in Supplementary Table S2. Additional supporting data are available from the corresponding author upon reasonable request.

## Supplementary Materials

The following supporting information can be downloaded at: https://www.mdpi.com/article/10.3390/biology15211948/s1, Figure S1: Uncropped original Western blot images of INHA and β-actin; Figure S2: Exploratory quantification of marker-associated fluorescence; Table S1: Sequences used for the *INHA* overexpression and knockdown experiments; Table S2: Primer sequences used for qRT-PCR and relative mRNA expression data; Table S3: Complete transcriptomic differential expression results, expression and analysis-input matrices, sample metadata, and sequencing quality metrics; Table S4: Complete proteomic differential abundance results, primary and sensitivity-analysis abundance matrices, and sample metadata; Table S5: Integrated transcriptomic–proteomic results, complete candidate rankings, analysis settings, and individual Western blot and flow-cytometry data with detailed statistical results.

## References

[1] Qiu Q., Zhang G., Ma T., Qian W., Wang J., Ye Z., Cao C., Hu Q., Kim J., Larkin D.M. (2012). The yak genome and adaptation to life at high altitude. Nat. Genet..

[2] Ayalew W., Chu M., Liang C., Wu X., Yan P. (2021). Adaptation Mechanisms of Yak (*Bos grunniens*) to High-Altitude Environmental Stress. Animals.

[3] Zhu Y., Li X., Zhaxi L., Zhaxi S., Suolang, Ciyang, Sun G., Yangji C., Wangdui B. (2022). House feeding pattern increased male yak fertility by improving gut microbiota and serum metabolites. Front. Vet. Sci..

[4] Sarkar M., Dutta Borah B.K., Bandopadhayay S., Meyer H.H.D., Prakash B.S. (2009). Season of the year influences semen output and concentrations of testosterone in circulation of yaks (*Poephagus grunniens* L.). Anim. Reprod. Sci..

[5] Wang X., Guo S., Xiong L., Wu X., Bao P., Kang Y., Cao M., Ding Z., Liang C., Pei J. (2024). Complete characterization of the yak testicular development using accurate full-length transcriptome sequencing. Int. J. Biol. Macromol..

[6] O’Donnell L., Smith L.B., Rebourcet D. (2022). Sertoli cells as key drivers of testis function. Semin. Cell Dev. Biol..

[7] Wang X., Pei J., Xiong L., Guo S., Cao M., Kang Y., Ding Z., La Y., Liang C., Yan P. (2023). Single-Cell RNA Sequencing Reveals Atlas of Yak Testis Cells. Int. J. Mol. Sci..

[8] Zimmermann C., Stévant I., Borel C., Conne B., Pitetti J.-L., Calvel P., Kaessmann H., Jégou B., Chalmel F., Nef S. (2015). Research Resource: The Dynamic Transcriptional Profile of Sertoli Cells During the Progression of Spermatogenesis. Mol. Endocrinol..

[9] Guo S., Pei J., Wang X., Cao M., Xiong L., Kang Y., Ding Z., La Y., Chu M., Bao P. (2023). Transcriptome Studies Reveal the N^6^-Methyladenosine Differences in Testis of Yaks at Juvenile and Sexual Maturity Stages. Animals.

[10] Zhang H., Liu B., Qiu Y., Fan J.F., Yu S.J. (2013). Pure cultures and characterization of yak Sertoli cells. Tissue Cell.

[11] Wang Y., Pan Y., Wang M., Afedo S.Y., Zhao L., Han X., Liu M., Zhao T., Zhang T., Ding T. (2022). Transcriptome sequencing reveals differences between leydig cells and sertoli cells of yak. Front. Vet. Sci..

[12] Hu L., Wang X., Guo S., Cao M., Kang Y., Ding Z., Pei J., Ge Q., Ma Y., Guo X. (2024). Whole-transcriptome sequencing analysis to identify key circRNAs, miRNAs, and mRNAs in the development of yak testes. BMC Genom..

[13] Goney M.P., Wilce M.C.J., Wilce J.A., Stocker W.A., Goodchild G.M., Chan K.L., Harrison C.A., Walton K.L. (2020). Engineering the Ovarian Hormones Inhibin A and Inhibin B to Enhance Synthesis and Activity. Endocrinology.

[14] Anderson R.A., Wallace E.M., Groome N.P., Bellis A.J., Wu F.C. (1997). Physiological relationships between inhibin B, follicle stimulating hormone secretion and spermatogenesis in normal men and response to gonadotrophin suppression by exogenous testosterone. Hum. Reprod..

[15] Lucas-Herald A.K., Mitchell R.T. (2022). Testicular Sertoli Cell Hormones in Differences in Sex Development. Front. Endocrinol..

[16] Lewis K.A., Gray P.C., Blount A.L., MacConell L.A., Wiater E., Bilezikjian L.M., Vale W. (2000). Betaglycan binds inhibin and can mediate functional antagonism of activin signalling. Nature.

[17] Nicholls P.K., Stanton P.G., Chen J.L., Olcorn J.S., Haverfield J.T., Qian H., Walton K.L., Gregorevic P., Harrison C.A. (2012). Activin Signaling Regulates Sertoli Cell Differentiation and Function. Endocrinology.

[18] Xu K., Wang J., Liu H., Zhao J., Lu W. (2020). Melatonin Promotes the Proliferation of Chicken Sertoli Cells by Activating the ERK/Inhibin Alpha Subunit Signaling Pathway. Molecules.

[19] Vogel C., Marcotte E.M. (2012). Insights into the regulation of protein abundance from proteomic and transcriptomic analyses. Nat. Rev. Genet..

[20] Upadhya S.R., Ryan C.J. (2022). Experimental reproducibility limits the correlation between mRNA and protein abundances in tumor proteomic profiles. Cell Rep. Methods.

[21] Ma R., Cui Y., Yu S.-J., Pan Y.-Y., He J.-F., Wang Y.-Y., Wang J.-L., Wang X.-Y., Bai X.-F., Zhang H. (2025). The glucose metabolism reprogramming of yak Sertoli cells under hypoxia is regulated by autophagy. BMC Genom..

[22] Chen S., Zhou Y., Chen Y., Gu J. (2018). fastp: An ultra-fast all-in-one FASTQ preprocessor. Bioinformatics.

[23] Langmead B., Salzberg S.L. (2012). Fast gapped-read alignment with Bowtie 2. Nat. Methods.

[24] Kim D., Paggi J.M., Park C., Bennett C., Salzberg S.L. (2019). Graph-based genome alignment and genotyping with HISAT2 and HISAT-genotype. Nat. Biotechnol..

[25] Soneson C., Love M.I., Robinson M.D. (2016). Differential analyses for RNA-seq: Transcript-level estimates improve gene-level inferences. F1000Research.

[26] Muzellec B., Teleńczuk M., Cabeli V., Andreux M. (2023). PyDESeq2: A python package for bulk RNA-seq differential expression analysis. Bioinformatics.

[27] Love M.I., Huber W., Anders S. (2014). Moderated estimation of fold change and dispersion for RNA-seq data with DESeq2. Genome Biol..

[28] Subramanian A., Tamayo P., Mootha V.K., Mukherjee S., Ebert B.L., Gillette M.A., Paulovich A., Pomeroy S.L., Golub T.R., Lander E.S. (2005). Gene set enrichment analysis: A knowledge-based approach for interpreting genome-wide expression profiles. Proc. Natl. Acad. Sci. USA.

[29] Makanji Y., Zhu J., Mishra R., Holmquist C., Wong W.P.S., Schwartz N.B., Mayo K.E., Woodruff T.K. (2014). Inhibin at 90: From Discovery to Clinical Application, a Historical Review. Endocr. Rev..

[30] Matzuk M.M., Finegold M.J., Su J.-G.J., Hsueh A.J.W., Bradley A. (1992). Alpha-inhibin is a tumour-suppressor gene with gonadal specificity in mice. Nature.

[31] Uhlmann F. (2016). SMC complexes: From DNA to chromosomes. Nat. Rev. Mol. Cell Biol..

[32] Fan J., Schiemer T., Vaska A., Jahed V., Klavins K. (2024). Cell via Cell Viability Assay Changes Cellular Metabolic Characteristics by Intervening with Glycolysis and Pentose Phosphate Pathway. Chem. Res. Toxicol..

[33] Xie L., Dai Z., Pang C., Lin D., Zheng M. (2018). Cellular glucose metabolism is essential for the reduction of cell-impermeable water-soluble tetrazolium (WST) dyes. Int. J. Biol. Sci..

[34] Kari S., Subramanian K., Altomonte I.A., Murugesan A., Yli-Harja O., Kandhavelu M. (2022). Programmed cell death detection methods: A systematic review and a categorical comparison. Apoptosis.

[35] Chen R., Wang F., Chen Y., Han D. (2022). Immune homeostasis and disorder in the testis—Roles of Sertoli cells. J. Reprod. Immunol..

[36] Li S.-Y., Kumar S., Gu X., DeFalco T. (2024). Testicular immunity. Mol. Asp. Med..

[37] Hayden M.S., Ghosh S. (2011). NF-κB in immunobiology. Cell Res..

[38] Di Persio S., Starace D., Capponi C., Saracino R., Fera S., Filippini A., Vicini E. (2021). TNF-α inhibits GDNF levels in Sertoli cells, through a NF-κB-dependent, HES1-dependent mechanism. Andrology.

[39] Xia W., Wong E.W.P., Mruk D.D., Cheng C.Y. (2009). TGF-β3 and TNFα perturb blood–testis barrier (BTB) dynamics by accelerating the clathrin-mediated endocytosis of integral membrane proteins: A new concept of BTB regulation during spermatogenesis. Dev. Biol..

[40] Washburn R.L., Dufour J.M. (2023). Complementing Testicular Immune Regulation: The Relationship between Sertoli Cells, Complement, and the Immune Response. Int. J. Mol. Sci..

[41] Washburn R.L., Hibler T., Kaur G., Dufour J.M. (2022). Sertoli Cell Immune Regulation: A Double-Edged Sword. Front. Immunol..

[42] Zhang X.-N., Tao H.-P., Li S., Wang Y.-J., Wu S.-X., Pan B., Yang Q.-E. (2022). *Ldha*-Dependent Metabolic Programs in Sertoli Cells Regulate Spermiogenesis in Mouse Testis. Biology.

[43] Smith L.B., Milne L., Nelson N., Eddie S., Brown P., Atanassova N., O’Bryan M.K., O’Donnell L., Rhodes D., Wells S. (2012). KATNAL1 Regulation of Sertoli Cell Microtubule Dynamics Is Essential for Spermiogenesis and Male Fertility. PLoS Genet..

[44] Wu S., Lv L., Li L., Wang L., Mao B., Li J., Shen X., Ge R., Wong C.K.C., Sun F. (2021). KIF15 Supports Spermatogenesis Via Its Effects on Sertoli Cell Microtubule, Actin, Vimentin, and Septin Cytoskeletons. Endocrinology.

[45] Battle A., Khan Z., Wang S.H., Mitrano A., Ford M.J., Pritchard J.K., Gilad Y. (2015). Impact of regulatory variation from RNA to protein. Science.

[46] Namwanje M., Brown C.W. (2016). Activins and Inhibins: Roles in Development, Physiology, and Disease. Cold Spring Harb. Perspect. Biol..

