# Supplementary material for: Integrated Transcriptomic and Proteomic Analyses Reveal Molecular and Cellular Responses to INHA Manipulation in Primary Yak Sertoli Cells
Source: Biology (Basel). 2026 Oct 29;15(21):1948. doi: 10.3390/biology15211948 (PMC13762315; doi:10.3390/biology15211948)
Supplement: Supplementary file 1 [file biology-15-01948-s001.zip › Supplementary Figures.pdf]

A

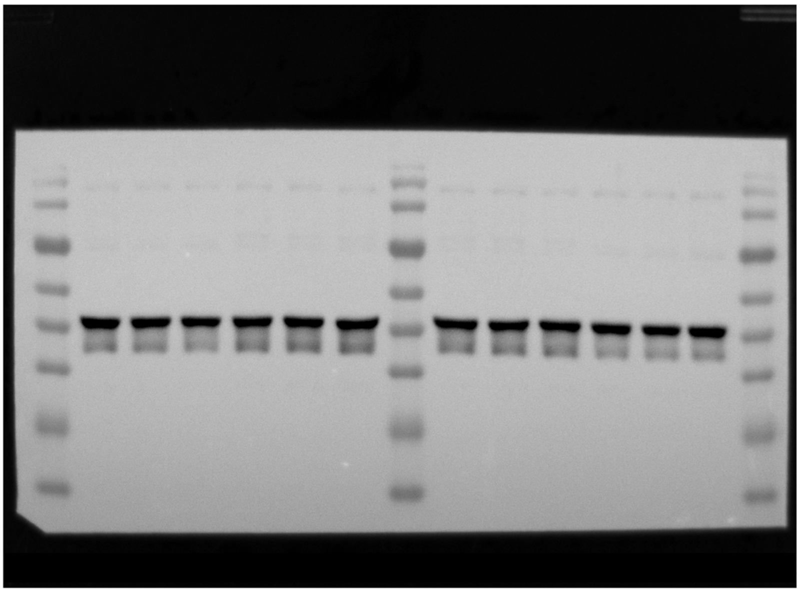

B

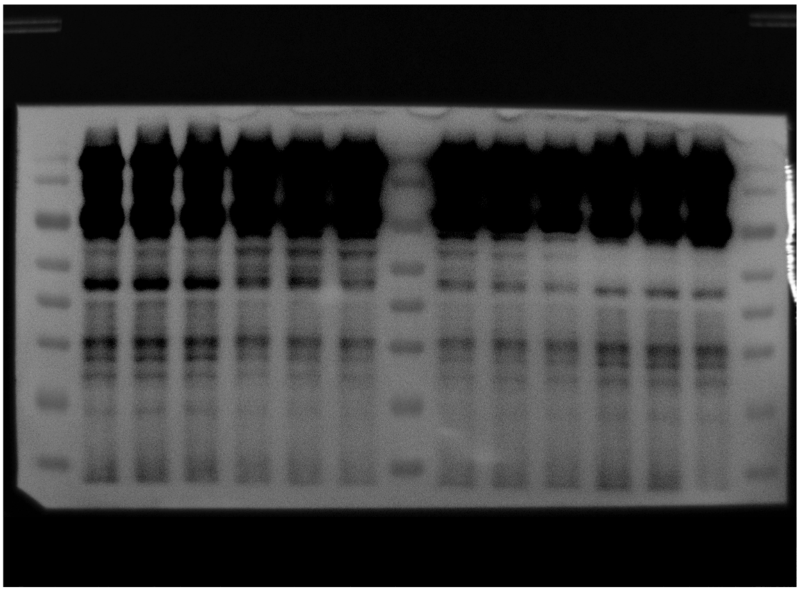

Figure S1. Uncropped Western blot images used for the analysis of INHA protein expression. (A) Uncropped blot for INHA. (B) Uncropped blot for β-actin, which was used as the loading control.

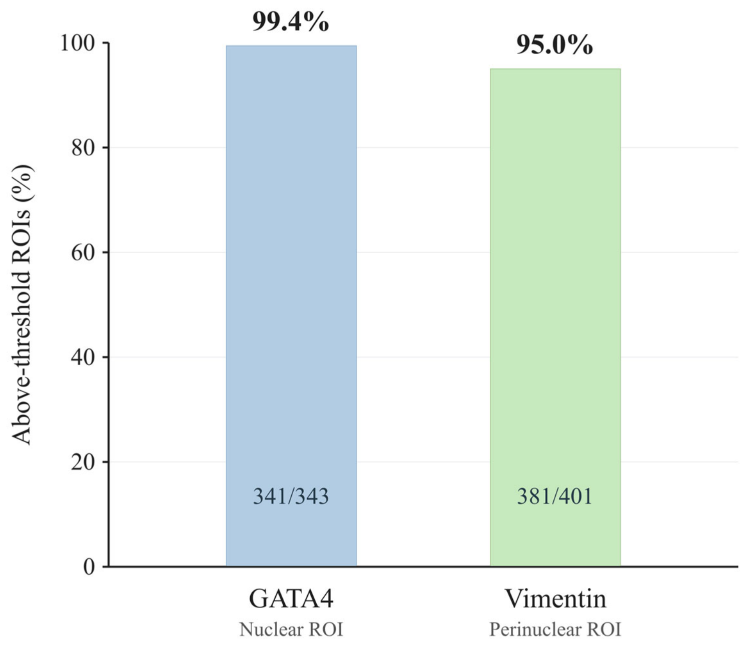

Figure S2. Exploratory quantification of marker-associated fluorescence. One field per stain was analyzed. Above-threshold fractions were 341/343 nuclear ROIs for GATA4 and 381/401 perinuclear ROIs for vimentin. Vimentin-associated signal was measured in regions extending up to 8 pixels outside nuclear boundaries. Spatial background was sampled more than 20 pixels from detected nuclei, excluding scale annotations. The cutoff was background median + k × 1.4826 × MAD, with k = 2 selected post hoc. Vimentin fractions were 89.0% and 76.3% at k = 3 and 4, respectively. These descriptive image-level signal fractions are not validated estimates of culture purity. MAD, median absolute deviation; ROI, region of interest.
